# Natural Variation of a PPR Coding Gene *SST1* Confers Salt Tolerance During Soybean Domestication

**DOI:** 10.1111/pbi.70382

**Published:** 2025-09-25

**Authors:** Hui Wang, Huifang Yuan, Yadi Wang, Weikang Xi, Xiaodi Wang, Ruiheng Tang, Qi Xu, Jiaying Li, Dongxu Liu, Qingyong Yang, Xutong Wang, Fanjiang Kong, Baohui Liu, Xia Li, Zhijuan Wang

**Affiliations:** ^1^ National key Laboratory of Crop Genetic Improvement, Hubei Hongshan Laboratory, College of Plant Science and Technology Huazhong Agricultural University Wuhan Hubei China; ^2^ National key Laboratory of Crop Genetic Improvement, Hubei Key Laboratory of Agricultural Bioinformatics, College of Informatics Huazhong Agricultural University Wuhan Hubei China; ^3^ Innovative Center of Molecular Genetics and Evolution, School of Life Sciences Guangzhou University Guangzhou China

**Keywords:** domestication, natural variation, salt tolerance, soybean

## Abstract

Soil salinity is one of the constraints that adversely affect seedling growth and limit soybean yield. Identifying salt tolerance genes and profiling their allele variants are crucial for elucidating the mechanisms underlying salt tolerance in soybean and enabling the genetic improvement of salt‐tolerant cultivars. Here, we developed a salt‐induced leaf senescence‐based screening system to assess salt tolerance and identified a key salt tolerance gene, *SST1*, which encodes a pentatricopeptide repeat (PPR) protein, via genome‐wide association analysis. We showed that the truncated allele *SST1* ^HapT^, which has a nonsense mutation, increases salt tolerance in soybean, whereas the full‐length allele *SST1* ^HapC^ does not. Located in mitochondria, SST1 regulates RNA editing of the mitochondrial genes *cob*, *nad3* and *atp6‐1*, thereby influencing mitochondrial morphology and H_2_O_2_ homeostasis in root cells. *SST1* is an adaptive domestication‐related gene; the truncated *SST1* ^HapT^ allele is exclusively fixed in cultivated soybean but absent in wild soybean, indicating selection under increasing soil salinity during domestication. Furthermore, our results revealed that *SST1* regulates salt tolerance by synergistic interaction with *GmCHX1*, a pivotal salt tolerance gene unselected during domestication. Our findings provide valuable insights into soybean domestication and offer targets for enhancing soybean salt tolerance.

## 1 Introduction

Soybean [ *Glycine max* (L.) Merr.] is an economically important legume crop with high protein and oil contents (Wang et al. 2020). Soybean has unique characteristics, such as nitrogen fixation through symbiosis, which increases soil fertility, making it an excellent rotational crop. The demand for soybean has been increasing over the years, but soybean production has not kept pace with this demand due to yield and land constraints. Specifically, the application of fertilisers and improper cultivation practices have led to severe environmental problems, including soil salinity (Tarolli et al. 2024). Soybeans are commonly classified as moderately salt‐sensitive crops (Munns and Tester 2008; Do et al. 2016). When the soil salinity exceeds 5 dS/m, a substantial loss in soybean yield occurs (Ashraf and Wu 1994). At soil salinity levels of 14–15 and 18–20 dS/m, the soybean yield decreased by 52.5% and 61.1%, respectively (Chang et al. 1994). Salt stress affects almost every process in the soybean plants life cycle. This type of stress inhibits seed germination, seedling growth and reproductive growth by negatively affecting various biological processes, including photosynthesis (Zhang et al. 2018), symbiotic nitrogen fixation (Singleton and Bohlool 1984; Elsheikh and Wood 1995) and water and nutrient uptake and utilisation (Xu et al. 2017). Under natural conditions, soybean seedlings show inhibited growth due to aggravated salt stress caused by rising temperatures and insufficient water, resulting in plant death and major yield losses (Chang et al. 1994). Excessive ions disrupt protein biosynthesis and metabolic processes and cause excessive production of reactive oxygen species (ROS), which results in cell damage and even death (Jones 2000; Mittler et al. 2022). Therefore, increasing soybean yield and salt tolerance is critical to meet the high demand for this crop. However, modern soybean breeding programmes are usually aimed at achieving high yields under normal soil conditions. Although physical and chemical management of soil can partially mitigate the adverse effects of soil salinity, genetic improvement to increase the salt tolerance of soybeans is a more fundamental strategy for achieving consistently high yields.

Salt tolerance is a trait involving multiple genes and processes. To date, several genes related to soybean salt tolerance have been identified viareverse genetics approaches. These factors include ion channel proteins (Li et al. 2006; Chen et al. 2014; Guan et al. 2014; Qi et al. 2014; Nie et al. 2015; Wei et al. 2016), such as Na^+^/H^+^ antiporter 1 (GmNHX1), which sequesters Na^+^ from the cytoplasm into the vacuole (Li et al. 2006), and transcription factors (Shi et al. 2018; Yang et al. 2020; Zhang et al. 2020; Li et al. 2021; Ke et al. 2022; Wang et al. 2022; Li, Zhu, et al. 2023; Zhang, Zhi, et al. 2023), such as the membrane‐bound NAC WITH TRANS‐MEMBRANE MOTIF1‐LIKE (GmNTL1), which regulates both ion transport and ROS production‐related genes (Zhang, Zhi, et al. 2023). Despite this progress (Li et al. 2006, 2021; Qi et al. 2014; Nie et al. 2015; Shi et al. 2018; Yang et al. 2020; Zhang et al. 2020; Wang et al. 2022; Li, Zhu, et al. 2023; Zhang, Zhi, et al. 2023), the large soybean genome and its abundant genetic variation suggest that there may be outstanding genetic loci yet to be discovered. Soybeans were domesticated in China approximately 6000–9000 years ago (Kim et al. 2012). During domestication and genetic improvement, the diversity of soybean underwent significant changes. Cultivation in diverse environments has led to the accumulation of critical genetic variants essential for adaptation to various growth conditions, including soil salinity. These variants represent valuable resources for genetic breeding. Thus, identifying salt‐tolerant soybean germplasms and genes is essential for elucidating the molecular mechanisms of salt tolerance and breeding superior salt‐tolerant varieties. Previous studies have demonstrated major genetic and salt tolerance differences between wild soybean ( *Glycine soja* ) and cultivated soybean ( *Glycine max* ) (Ha et al. 2013; Qi et al. 2014; Zhou et al. 2015), as well as among soybean germplasms (Zhang et al. 2014; Kan et al. 2015, 2016). For example, Guan et al. identified two alleles (H1 and H7) of the *GmCHX1* gene in salt‐tolerant soybean varieties, which exhibited minimal genetic variation. In contrast, salt‐sensitive soybean varieties exhibited greater genetic diversity, with seven distinct alleles (H2–H6, H8 and H9). Additionally, the H6–H9 haplotypes of the *GmCHX1* gene were exclusively found in wild soybean, while the H1, H4, and H5 haplotypes of the *GmCHX1* gene were predominantly found in cultivar soybean (Guan et al. 2014). Notably, a stable major quantitative trait locus (QTL) has been repeatedly detected on chromosome 3 in soybean (Chen et al. 2008; Hamwieh and Xu 2008; Hamwieh et al. 2011; Guan et al. 2014; Do et al. 2016; Patil et al. 2016). This major QTL related to salt tolerance at the soybean seedling stage could explain 41% and 60% of the phenotypic variation in salt tolerance between S‐100 (salt‐tolerant) and Tokyo (salt‐sensitive) soybeans in field and greenhouse experiments, respectively, and two molecular markers, Sat_091 and Satt237, were identified for salt tolerance in soybean breeding (Lee et al. 2004). Further comparative resequencing of the salt‐tolerant wild soybean parent W05 and the salt‐sensitive parent C08 led to the identification of a cation/H^+^ antiporter (*GmCHX1*) as the key gene conferring salt tolerance (Qi et al. 2014). The *Ty1*/*copia* transposon insertion in the third exon of the *GmCHX1* gene causes a truncated transcript to disrupt its transporter function, thereby resulting in reduced salt tolerance in soybean plants (Qi et al. 2014). *GmCHX1/*(*salt tolerance‐associated gene on chromosome 3*, *GmSALT3*) was also identified through map‐based cloning, and GmCHX1/GmSALT3 is located in the endoplasmic reticulum and predominantly regulates ion transport in roots (Guan et al. 2014). In addition, several QTLs related to salt tolerance have been identified in soybean. For example, using 132 F_2_ progenies of the salt‐tolerant soybean Fiskeby III (PI 438471) with the salt‐sensitive soybean Williams 82 (PI 518671), Tuyen et al. identified a candidate salt‐tolerant QTL on chromosome 13 (Do et al. 2018). Recently, several salt‐tolerant loci have been identified via genome‐wide association studies (GWASs) in soybean, and the natural variations in two genes, *cation diffusion factor* (*GmCDF1*) and *early responsive to dehydration 15B* (*GsERD15B*), are related to salt tolerance during seed germination and seedling growth, respectively (Zhang et al. 2019; Jin et al. 2021). Two natural variations in GmCDF1 are associated with salt tolerance by affecting K^+^/Na^+^ homeostasis (Zhang et al. 2019), and a 7‐bp deletion in the *GsERD15B* promoter increases soybean plant salt tolerance through the modulation of abscisic acid (ABA) signalling and ion uptake (Jin et al. 2021). These results indicate that GWAS is a useful way to identify salt tolerance genes and natural variations that can be used for the molecular breeding of elite varieties with increased salt tolerance. However, research to identify salt tolerance genes and variations is still in its infancy. A rapid and effective evaluation system for the salt tolerance of soybean at various stages and extensive identification of associated salt tolerance genes and natural variations will facilitate our understanding of the molecular basis of salt tolerance in soybean during evolution and breeding.

In this study, we used salt‐induced leaf senescence (SILS) as an indicator to evaluate salt tolerance across a natural population of diverse soybean accessions. Through GWAS, we identified a variant at the *Glyma.09G000400* locus, designated as *soybean salt tolerant 1* (*SST1*), on chromosome 9. *SST1* encodes a pentatricopeptide‐repeat (PPR) protein, a nucleus‐encoded member of the PPR family characterised by multiple tandem repeats of a degenerate 35‐amino‐acid motif and a PPR motif. These proteins play crucial roles in plant growth and development and abiotic tolerance (Mochizuki et al. 1996; Zsigmond et al. 2008; Liu et al. 2010, 2016; Laluk et al. 2011; Murayama et al. 2012; Yuan and Liu 2012; Barkan and Small 2014; Zhu et al. 2014; Jiang et al. 2015; Wu et al. 2018; Qiu et al. 2021). PPR proteins are sequence‐specific RNA‐binding proteins that are mostly localised in mitochondria and/or chloroplasts, where post‐transcriptional processing of RNA occurs (Barkan and Small 2014). In model plants, such as *Arabidopsis* and rice, PPR proteins have a range of functions in the post‐transcriptional processes of mitochondria and chloroplasts, including RNA editing, RNA splicing, RNA cleavage and translation (Schmitz‐Linneweber and Small 2008). Some PPR genes mediate plant stress tolerance by regulating the balance of mitochondrial ROS in response to abiotic and biotic stress (Zsigmond et al. 2008; Liu et al. 2010, 2016; Laluk et al. 2011; Murayama et al. 2012; Yuan and Liu 2012; Zhu et al. 2014; Jiang et al. 2015; Qiu et al. 2021). However, the function of PPR proteins in the salt tolerance of soybean has not been studied. Here, we found that a nonsense variation in *SST1* produces the allele *SST1* ^HapT^, encoding a truncated peptide that is associated with increased salt tolerance in soybean accessions. In addition, the transcription of *SST1* is not responsive to salt stress, but the SST1 protein level in mitochondria is significantly reduced in response to salt stress. Moreover, *SST1* is an adaptive domestication‐related gene with a truncated allele of *SST1* that is exclusively fixed in cultivated soybean rather than wild soybean, and intriguingly, *SST1* appears to regulate salt tolerance in a *GmCHX1*‐independent mechanism. These findings indicate that *SST1* plays an important role in wild soybean's adaptation to saline environments and represents a valuable genetic target for breeding salt‐tolerant soybean cultivars.

## 2 Results

### 2.1 Identification of Salt Tolerant Loci in Soybean Using GWAS

**FIGURE 1:**
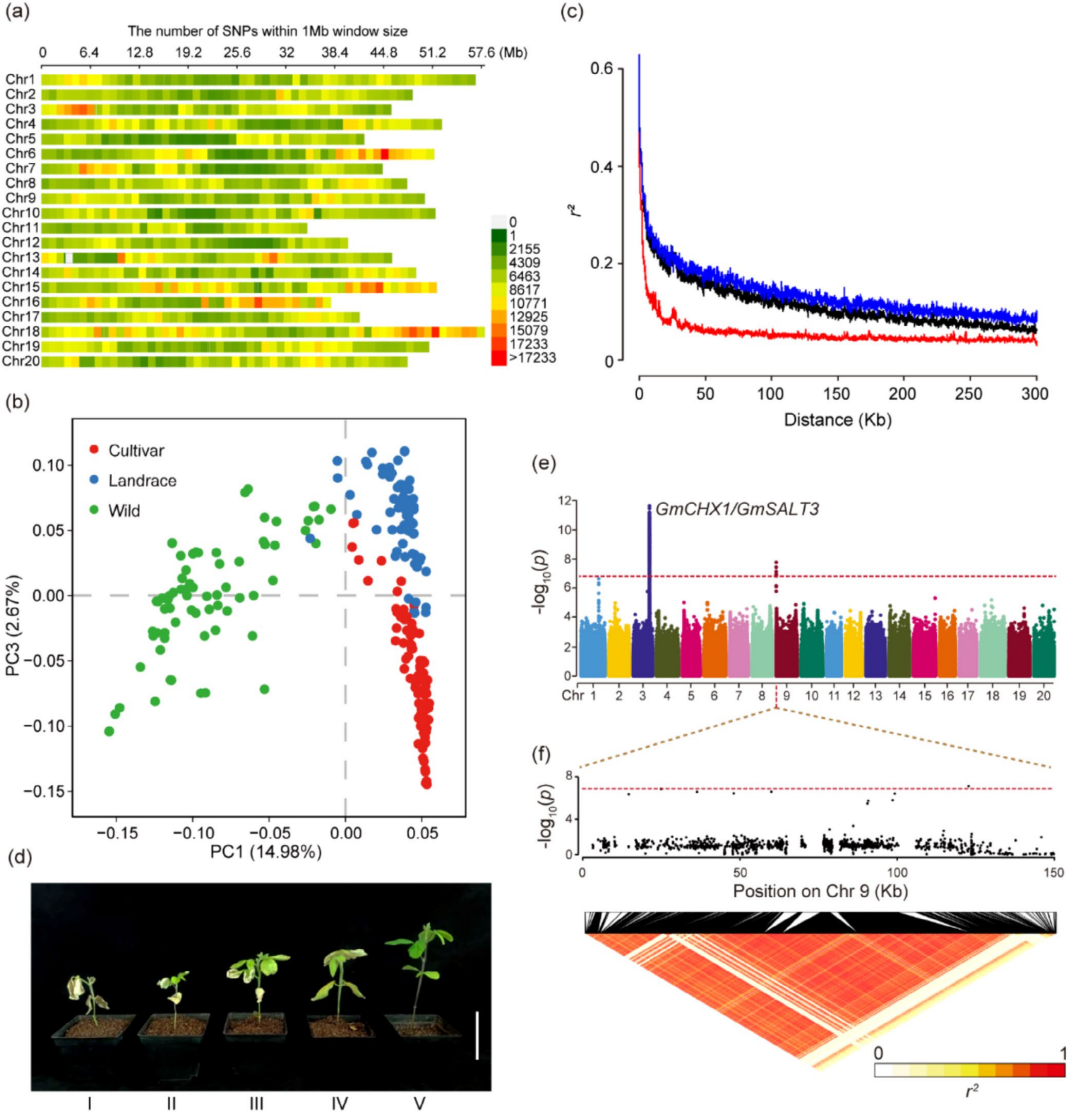
Characterisation and GWAS for salt tolerance in soybean. (a) SNP density along 20 chromosomes. The colour indicates the number of SNPs in a 1‐Mb region. (b) PCA of genetic diversity within the panel. (c) Linkage disequilibrium decay for the three subgroups within the diversity panel. Linkage disequilibrium decay is determined by squared correlations of allele frequencies (*r* ^2^) against the distance between polymorphic sites in wild soybeans (red), landraces (black) and cultivars (blue). (d) Representative images of 5 levels of SILS. Bar = 5 cm. (e) Association analysis of SILS. The red lead SNPs are shown above the threshold signals. (f) LD analysis of SILS candidate SNP loci. Upper panel showed the genome‐wide Manhattan plot in the 0–117.19 kb region on chromosome 9. The red lead SNPs are shown above the threshold signals. Lower panel showed the pairwise *r* ^2^ values among all polymorphic sites in the genomic region (0–117.19 kb).

To identify key genes and genetic variations associated with salt tolerance across diverse genetic backgrounds, we selected 240 core soybean accessions from various regions of China (Li, Sun, et al. 2023). The alignment comparison with the soybean reference genome (Williams 82.a2, PI 518671) followed by variant calling, low‐quality variant filtering and genotype imputation identified a total of 6 772 053 high‐quality biallelic SNPs, with 1 SNP per 153 bp on average (Figure 1a), and 807 459 insertions/deletions (INDELs). Principal component analysis (PCA) of genome‐wide SNP markers revealed three distinct genetic clusters among the accessions (wild soybeans, landraces and improved cultivars) (Figure 1b, Table S1), consistent with previously reported population structures in soybean. The linkage disequilibrium (*r* ^2^) decreased with physical distance between SNPs, allowing the mapping of associations to relatively narrow genomic regions (Figure 1c).

The salt tolerance of crops is ultimately determined by the yield from the field (Flowers 2004). Because of the substantial difficulty in evaluating crop performance under saline conditions, crop yield is not commonly used to evaluate crop salt tolerance (Flowers 2004). To evaluate soybean germplasms under salt stress, SILS was used as a phenotypic indicator (Figure 1d). The results revealed significant differences in salt tolerance across soybean germplasms (Figure 1d, Figure S1a). The degree of tolerance was scored on a scale from 1 (hypersensitivity) to 5 (salt tolerance) (Figure S1a).

A mixed linear model‐based GWAS identified a major QTL peak on chromosome 3 and a secondary QTL peak on chromosome 9 (Figure 1e). The highest peak is located with the well‐known soy salt stress‐related gene *GmCHX1* (also known as *GmSALT3*) (Guan et al. 2014; Qi et al. 2014), suggesting that the index systems for the evaluation of soybean salt tolerance are reliable and feasible. In addition, we generated a quantile–quantile (QQ) plot to assess the reliability of our GWAS results. The observed *p*‐values closely followed the expected distribution, particularly in the lower quantiles (first half of the plot), where they aligned nearly parallel to the diagonal without significant deviation (Figure S1b). This suggests that there are no significant false positives or false negatives in the results of GWAS. Next, the candidate loci within the 117.19 kb region spanning 10 genes on chromosome 9 were analysed (Figure 1e). To accurately identify candidate genes, we used a strict *p* value threshold (*p* < 1.48 × 10^−7^) to select potential candidate SNPs in coding regions of these genes. A total of 10 suggestive SNPs (*p* < 1 × 10^−5^) significantly associated with salt tolerance were identified within the target interval on chromosome 9 (Figure 1f). These SNPs spanned three candidate genes: *Glyma.09G000400*, *Glyma.09G000700* and *Glyma.09G001200*. The remaining SNP loci in the region showed weaker associations (*p* > 1 × 10^−4^) (Table S2).

To identify key genes involved in soybean salt tolerance, we first examined the transcript abundance of the three candidate genes in response to salt stress using randomly selected salt‐tolerant and salt‐sensitive accessions. There were no significant changes in the transcript abundance of the three candidate genes under either short‐term (3 h) or long‐term (5 days) salt stress treatment (Figure S2), suggesting that these genes are not responsive to salt stress at the transcriptional level. To investigate the role of these three genes in salt tolerance, we performed overexpression assays in soybean cultivar Williams 82 (W82) using hairy root transformation technology and evaluated their effects on salt tolerance. Intriguingly, compared to empty vector (EV), only composite plants overexpressing *Glyma.09G000400* exhibited increased salt sensitivity, as evidenced by significantly lower SILS scores, whereas plants overexpressing *Glyma.09G000700* and *Glyma.09G001200* showed no significant differences in salt tolerance relative to EV controls (Figure S3). This result suggests that *Glyma.09G000400* is the causal gene that underlies variation in salt tolerance. We then named *Glyma.09G000400* as *SST1* for further analyses.

Previous studies have reported assembly quality issues in certain regions of the Williams 82.a2 genome. Given these concerns, we also realigned the resequenced raw data with the higher‐quality Gmax_ZH13 reference genome assembled by Shen et al. (2018) and conducted a comprehensive GWAS analysis. The same QTL locus was identified on chromosome 9 in ZH13 genomes (Figure S4). Further analysis showed that *SoyZH13_09G000500* in the ZH13 genome corresponds to *Glyma.09G000400* in the Williams 82.a2 genome, and a significant SNP (C‐to‐T) at position 697 of *SoyZH13_09G000500* exhibited a *p*‐value of 7.5128 × 10^−9^, exceeding the significance threshold (Table S2). There was a highly significant correlation between the 697th base of *SST1* and soybean salt tolerance across two reference genomes, indicating that the variation in the 697th base of *SST1* is critical for soybean salt tolerance.

We also performed SV‐GWAS (structural variation GWAS) on soybean salt tolerance using the MLM model in the ZH13 genome. While the *GmCHX1* locus showed a significant association in the MLM analysis, SV‐GWAS yielded fewer candidate SVs with weaker statistical support compared to SNP/InDel‐based GWAS (Figure S5). These results demonstrate that SVs contribute to GmCHX1‐mediated salt tolerance, in addition to SNPs and InDels. The combined evidence suggests that GmCHX1 harbours diverse and extensive genetic variation influencing salt tolerance.

### 2.2 SNP24932 Causes Premature Termination of the *SST1* Transcript

**FIGURE 2:**
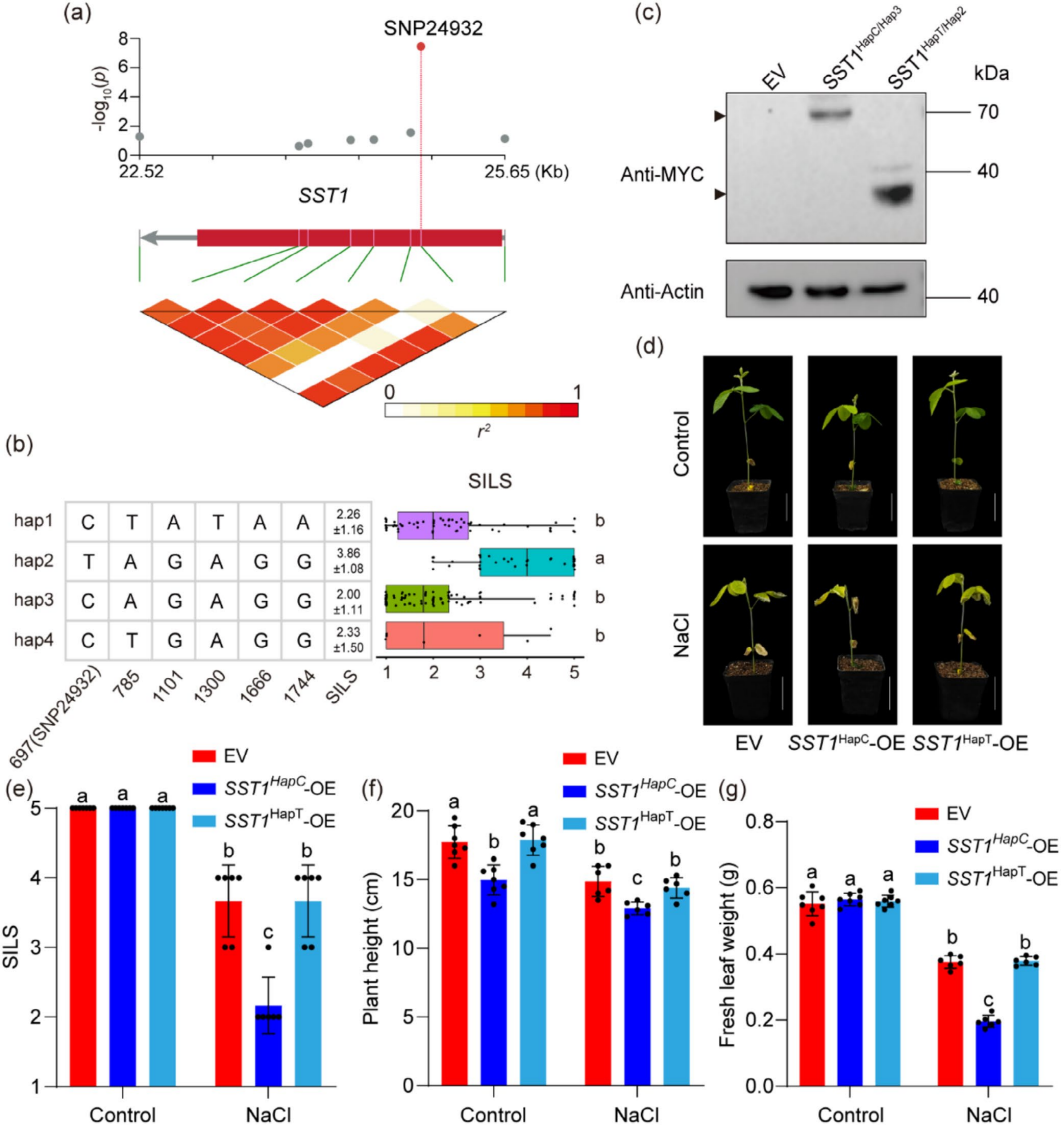
SNP24932 causes premature termination of the *SST1* transcript and the loss of function of *SST1*. (a) The association analysis of the genetic variations in *SST1* with SILS among 240 soybean natural germplasms. The upper panel showed the characterisation of variations significantly associated with SILS. The red dots highlighted SNP24932 that showed the highest association. The lower panel displayed the pattern of the pairwise *r* ^2^ values of the variations. (b) The left panel showed the haplotypes of *SST1* grouped according to the significant variants. Right panel showed the distributions of SILS for each haplotype group. Different letters indicate statistically significant differences at *p* < 0.05 by two‐way ANOVA test. (*n* = 61 for Hap1; *n* = 39 for Hap2; *n* = 92 for Hap3; *n* = 7 for Hap4). (c) Comparison of the protein levels of SST1 between HapC/Hap3 and HapT/Hap2. Actin was used as a control. The black arrows point to SST1^HapC/Hap3^ and SST1^HapT/Hap2^. (d) Soybean salt stress phenotypes in *SST1* ^HapC^ and *SST1* ^HapT^ transgenic soybean hairy roots. Bars = 5 cm. (e, f, g) Comparison of SILS, plant height and fresh leaf weight of transgenic soybean hairy roots of *SST1* ^HapC/Hap3^ and *SST1* ^HapT/Hap2^ under control and NaCl (100 mM) conditions. Data shown are the means ± SD of three independent experiments. Different letters indicate statistically significant differences at *p* < 0.05 based on two‐way ANOVA test (*n* = 7 for control; *n* = 6 for 100 mM NaCl).

To gain a deeper understanding of the functions of *SST1* in salt tolerance, we first conducted bioinformatic analyses of its gene and protein. *SST1* has only one exon (Figure S6a) and encodes a PPR family protein (871 amino acids) belonging to the PLS subclass of the PPR superfamily, with a DYW domain at its C‐terminus (Figure S6b,c). To determine the molecular basis of the functional variation in *SST1*, we analysed the associations of all variants of *SST1* with salt tolerance levels in soybean, and the results indicated that one SNP (SNP24932) was the most strongly associated with salt tolerance levels (Figure 2a). The linkage disequilibrium (*r* ^2^) block containing the lead SNP, SNP24932 (*p* = 3.53 × 10^−8^), is located in the CDS of *SST1* (Figure 2a). To more accurately analyse the haplotype of *SST1*, we removed the heterozygous sites of *SST1* from 240 natural soybean accessions, and the final 199 natural soybean accessions were divided into four haplotype groups (Hap1, Hap2, Hap3 and Hap4) (Figure 2b). The Hap1, Hap2, Hap3 and Hap4 groups were composed of 61, 39, 92, and 7 natural soybean accessions, respectively, with the Hap2 group conferring significantly greater salt tolerance levels than the Hap1, Hap3 and Hap4 groups (Figure 2b). Therefore, Hap2 was designated as the high‐salt‐tolerance haplotype, and Hap1, Hap3 and Hap4 were designated as the low‐salt‐tolerance haplotypes.

Further studies revealed that SNP24932 is a C‐to‐T variation at the 697th base of the exon of *SST1*, leading to a stop codon for the T variant, which produces a truncated protein of 232 amino acids. The western blot results revealed that HapC/Hap3 of *SST1* produced a full‐length SST1 protein with a predicted molecular weight of 70 kDa, whereas HapT/Hap2 produced a truncated protein of only 26 kDa (Figure 2c), indicating that the functional variation in SST1 is caused by the early termination of the protein. To confirm that the truncated *SST1* caused by SNP24932 truly lost its original function, the transcript of *SST1* ^HapC/Hap3^ and *SST1* ^HapT/Hap2^ was overexpressed in hairy roots of Changnong 17 (the haplotype is *SST1* ^HapT/Hap2^), and the salt response of these composite plants was evaluated (Figure S7). Indeed, compared with the expression of the EV, the overexpression of *SST1* ^HapC/Hap3^ resulted in a lower SILS, shorter plant height and lighter fresh leaf weight under salt stress, whereas the overexpression of *SST1* ^HapT/Hap2^ did not significantly affect salt tolerance (Figure 2d–g). Collectively, these results demonstrate that SNP24932 in the exon of *SST1* results in premature termination of its transcript, causing protein dysfunction and increasing salt tolerance in soybean germplasms.

### 2.3 *SST1* is a Negative Regulator of Salt Tolerance

**FIGURE 3:**
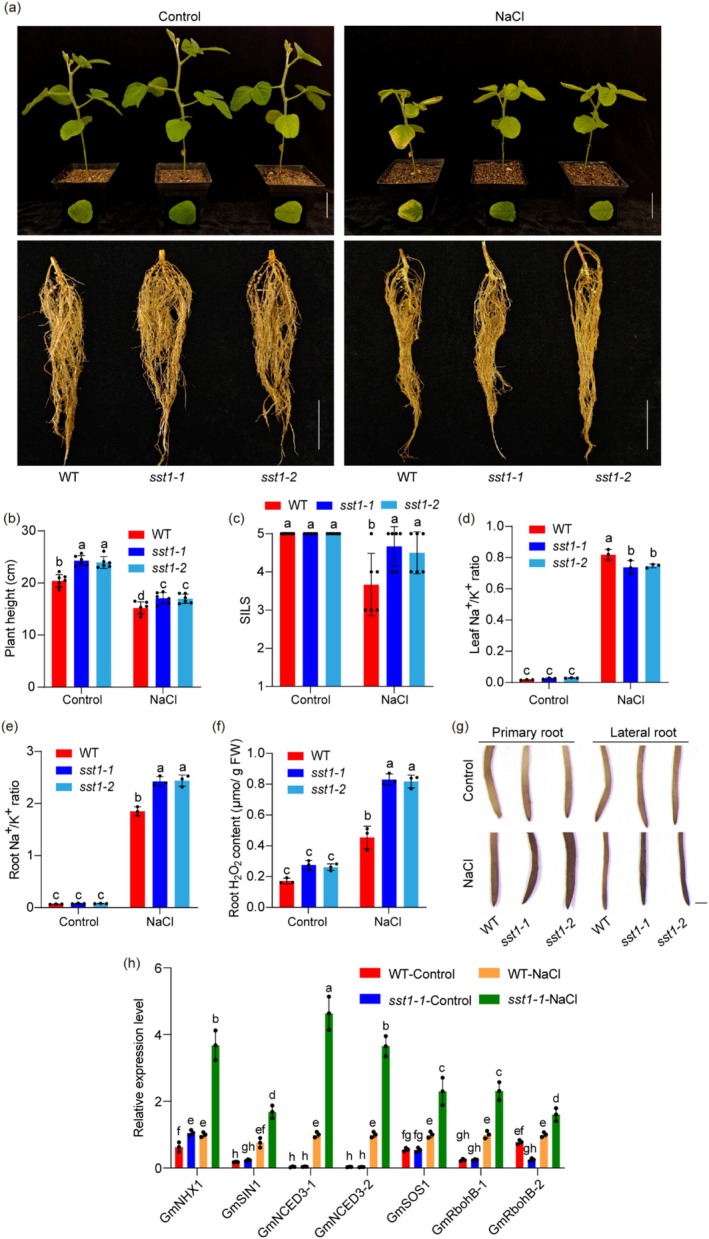
Salt tolerance phenotypes of *sst1* mutants under salt conditions and the expression levels of salt stress‐related genes involved in salt tolerance. (a) Phenotypes of WT, *sst1‐1* and *sst1‐2* under control and NaCl (100 mM) conditions. The upper panel showed the shoot phenotypes of WT, *sst1‐1* and *sst1‐2* and the lower panel showed the root phenotypes of WT, *sst1‐1* and *sst1‐2*. (b–f) Comparison of plant height, SILS, leaf Na^+^/K^+^ ratio, root Na^+^/K^+^ ratio and root H_2_O_2_ content of WT, *sst1‐1* and *sst1‐2* under control and NaCl (100 mM) conditions. Different letters indicate statistically significant differences at *p* < 0.05 by two‐way ANOVA test (*n* = 7 for WT at control; *n* = 6 for WT at 100 mM NaCl and *sst1* mutants). (g) DAB staining reveals the H_2_O_2_ content of the root of WT, *sst1‐1* and *sst1‐2* under control and NaCl (100 mM) conditions. The staining intensity reflects the concentration of H_2_O_2_. (h) The expression levels of salt stress‐related genes involved in salt tolerance. Data shown are the means ± SD of three independent experiments. Different letters indicate statistically significant differences at *p* < 0.05 based on the two‐way ANOVA test. *n* = 3.

To further confirm the role of *SST1* in soybean salt tolerance, the CRISPR/Cas9 technology was explored to generate two *SST1* knockout mutants in the W82 background (the haplotype is *SST1* ^HapC/Hap3^), and there was 1‐bp and a 4‐bp deletion in the coding region of *SST1* in *sst1‐1* and *sst1‐2*, respectively (Figure S8). Under control conditions, there were no significant differences in the fresh weight of leaves and roots, the Na^+^ and K^+^ contents, Na^+^/K^+^ ratio in leaves and roots, or the H_2_O_2_ content in roots between the wild‐type (WT) and *sst1* mutants, but the plant height in *sst1* mutants was significantly greater than in WT (Figure 3a–g, Figure S9). In contrast, under 100 mM NaCl conditions, both *sst1‐1* and *sst1‐2* presented significantly increased salt tolerance compared with the WT, with substantially increased SILS (Figure 3a,c). However, the fresh leaf weight and fresh root weight of the *sst1‐1* and *sst1‐2* mutants were comparable to those of the WT (Figure S9a,b). Interestingly, the *sst1* mutants exhibited distinct Na^+^ and K^+^ accumulation patterns: while leaf Na^+^ content increased, root Na^+^ remained unchanged. Conversely, leaf K^+^ content rose, whereas root K^+^ declined (Figure S9c–f). Consequently, the leaf Na^+^/K^+^ ratio in mutants was significantly lower than in WT, while the root Na^+^/K^+^ ratio showed a significant increase compared to WT roots (Figure 3d,e). These results indicate that SST1 regulates salt tolerance by modulating ion uptake and transport from the roots to the leaves. Proline and soluble sugars serve as key osmoprotectants that enhance plant stress tolerance, while malondialdehyde (MDA) serves as a biomarker for oxidative stress damage (Cao et al. 2007; Rosa et al. 2009; Jahantigh et al. 2016). Under control conditions, there were no significant differences in proline, soluble sugar, or MDA levels between WT and *sst1* mutants. However, under salt stress conditions, *sst1* mutants accumulated significantly higher levels of proline and soluble sugars, while exhibiting lower MDA content compared to WT (Figure S9g–i). In addition, H_2_O_2_ content in the root and leaf of the mutant plants was substantially greater than that of the WT plants under salt stress (Figure 3f,g; Figure S9j,k), suggesting that SST1 also mediates soybean salt tolerance by regulating H_2_O_2_ homeostasis. In order to simulate real field saline soil, we applied salt concentration gradients of 0‰, 3‰, 6‰ and 9‰ under greenhouse conditions and investigated the SILS, SPAD, plant height and number of trifoliate leaves of WT and *sst1‐1*. We found that *sst1‐1* is more tolerant to salt concentrations of 3‰ and 6‰, while WT is more sensitive (Figure S10). Specifically, in the V2 stage, both WT and *sst1‐1* can tolerate salt concentrations of 3‰, with no significant differences in their SILS, SPAD, and plant height (Figure S10a–d). Additionally, *sst1‐1* has a higher number of triple compound leaves (Figure S10e); however, *sst1‐1* is more tolerant to salt concentrations of 6‰, and its SILS and SPAD are significantly higher than in WT (Figure S10a–c); at a salt concentration of 9‰, all plants have died (Figure S10a–e). In the V4 stage, *sst1‐1* exhibited more pronounced salt tolerance characteristics at salt concentrations of 3‰ and 6‰. Specifically, under 3‰ and 6‰ salt treatment conditions, SILS, SPAD, plant height and the number of triple compound leaves in *sst1‐1* are higher than WT (Figure S10f–i); at a salt concentration of 9‰, all plants died (Figure S10a). Additionally, we examined the role of SST1 in salt tolerance during symbiotic nitrogen fixation. Unexpectedly, there were no significant differences in the number of nodules, nodule weight, or nitrogenase activity between the WT and *sst1* mutants (Figure S11). These results suggest that *SST1* specifically regulates salt tolerance rather than symbiotic nitrogen fixation in soybean plants.

To further investigate whether SST1 regulates salt tolerance through ion uptake and transport, we first analysed the expression of related genes, such as *GmNHX1* and *GmSOS1*, in WT and *sst1‐1* mutant. Under normal conditions, the transcript level of *GmNHX1* in the mutant was slightly greater than that in the WT, whereas the *GmSOS1* expression in the mutant was comparable to that in the WT. In sharp contrast, the expression of both genes was induced by salt stress, but the expression levels of the two genes in the mutant were dramatically greater than those in the WT (Figure 3h). These results suggest that SST1 inhibits ion uptake and transport by negatively regulating both genes. A previous study revealed that *GmSIN1*‐*GmNCED3* and *GmRbohBs* can form a positive feedback loop to regulate soybean salt tolerance through the rapid accumulation of ABA and ROS (Li et al. 2019). Because loss of function in *SST1* significantly increased ROS contents in the roots of the mutants, we next examined the expression of the *GmSIN1*, *GmNCED3‐1*, *GmNCED3‐2*, *GmRbohB‐1* and *GmRbohB‐2* genes. The expression levels of all these genes were induced by salt stress; however, *GmSIN1* and all the *GmNCED3s* and *GmRbohB* genes were up‐regulated in the *sst1‐1* mutant (Figure 3h). These results indicate that SST1 also regulates salt tolerance by affecting ABA biosynthesis and ROS production.

Given the extensive size of the PPR gene family, additional *SST1* homologues may contribute to salt tolerance. To investigate potential functional redundancy among *SST1* homologues in soybean, we employed CRISPR/Cas9‐mediated hairy root transformation to generate knockout lines targeting two SST1 paralogs (*Glyma.17G220100* and *Glyma.17G262700*, referred to as *G1* and *G2*) simultaneously and compared their phenotypes with *SST1* single gene knockout lines. Phenotypic analysis revealed a hierarchical salt tolerance response: *SST1* single knockout lines exhibited the strongest tolerance (evidenced by highest SILS scores and leaf fresh weight), while the *G1*/*G2* double knockout lines showed intermediate tolerance between EV and *SST1*‐KO lines (Figure S12). This result suggests that *SST1* has a stronger effect on salt tolerance than its paralogs for salt tolerance.

### 2.4 SST1 Is Localised in Mitochondria and Responds to Salt Stress at the Protein Level

**FIGURE 4:**
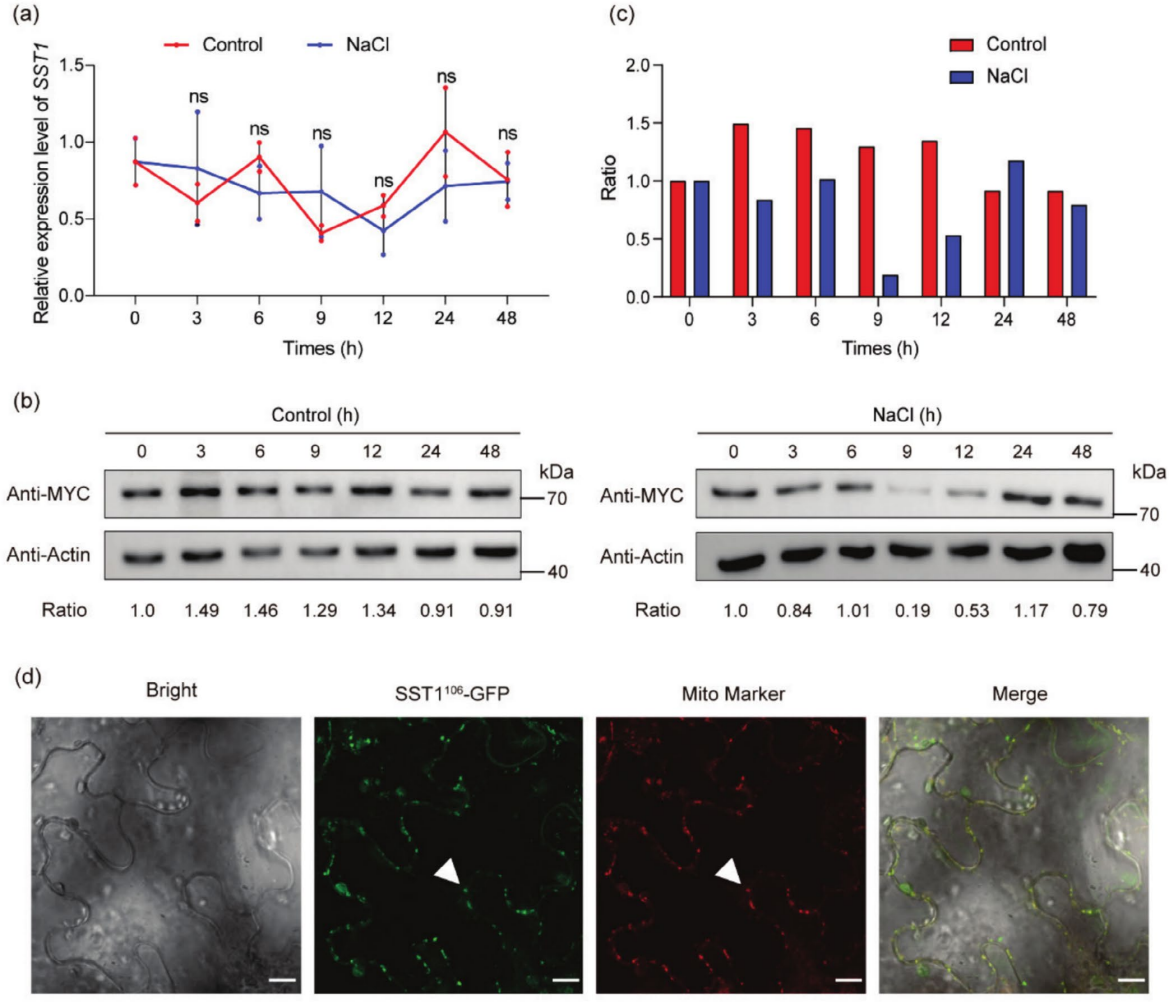
*SST1* Expression patterns at transcription and protein levels and subcellular localization of SST1. (a) Fourteen‐day‐old seedlings of wild type (WT) were treated under control and NaCl (100 mM) conditions and root materials were collected at 0, 3, 6, 9, 12, 24 and 48 h after treatment to detect transcriptional level expression. *GmELF1b* was used as the endogenous control gene. Statistically significant differences at *p* < 0.05 were analysed using a two‐sided *t*‐test. *n* = 3. (b) Twenty‐one‐day old *35S:SST1* Transgenic hairy roots were treated with or without NaCl (100 mM) and root materials were collected at 0, 3, 6, 9, 12, 24 and 48 h after treatment to detect protein level expression. Actin was used as a control. (c) Western blot grayscale analysis of *SST1*. Protein level quantification of SST1 is determined by band densitometry. (d) Confocal microscopic images showing co‐localization of the SST1^106^‐GFP and Mito‐Maker‐mCherry. Bars = 10 μm. The white arrows point to the mitochondria. Data shown are the means ± SD of three independent experiments.

To explore the underlying mechanism by which SST1 regulates salt tolerance in soybean, we first analysed the response of SST1 to salt stress at both the transcriptional and protein levels. Fourteen‐day‐old soybean seedlings of W82 were subjected to salt stress (100 mM NaCl), and the expression of *SST1* in roots and leaves was examined at the specified time points. The results revealed that *SST1* was expressed in both leaves and roots under normal conditions, but there was no significant change in the *SST1* transcript level in either tissue when treated with salt within 48 h (Figure 4a; Figure S13). Further promoter analysis revealed multiple *cis* elements related to the response to abiotic stress within the 3 kb promoter region of *SST1* (Figure S14). Next, the tissue/cell expression patterns of *SST1* were examined in transgenic hairy roots expressing *proSST1:GUS* in W82. There was no significant difference in GUS activity between transgenic hairy roots under normal and salt treatment conditions (Figure S15). These results indicate that *SST1* does not respond to salt stress at the transcriptional level. To determine whether the SST1 protein is affected by salt stress, we generated hairy roots overexpressing the SST1‐Myc fusion protein under the control of the CaMV35S promoter and measured the protein level via western blot under salt stress at different time points. Interestingly, the results revealed a dynamic response of the SST1 protein to salt stress. The SST1 protein level substantially decreased, reaching the lowest level at 9 h after exposure to salt stress, and then gradually returned to the original level at 24 h after salt treatment (Figure 4b,c). These results indicate that SST1 responds to salt stress and regulates plant salt tolerance at the protein level.

Many PPR proteins are localised in organelles to mediate post‐transcriptional regulation of their functions (Barkan and Small 2014). To further investigate the SST1 protein, we performed iPSORT prediction analysis. The results revealed that the SST1 protein has a mitochondrion‐targeting signal (27 amino acids) at its N‐terminus (Figure S16), suggesting that SST1 may be localised in mitochondria. To test this possibility, an N‐terminal SST1 fragment (Met1–Trp106) was fused with GFP (SST1^106^‐GFP) and then transformed into the epidermal cells of *Nicotiana benthamiana* leaves. When SST1^106^‐GFP was transiently expressed in the epidermal cells of *N. benthamiana* leaves, the green fluorescent signal of SST1^106^‐GFP was observed overlapping with the signals from the mitochondrial marker (Figure 4d). These results suggest that SST1 is a mitochondrial protein and may be involved in the post‐transcriptional regulation of mitochondria.

### 2.5 SST1 Affects the RNA Editing of *Cob*, *nad3* and *atp6‐1* and Mitochondrial Morphology in Soybean Root Cells

**FIGURE 5:**
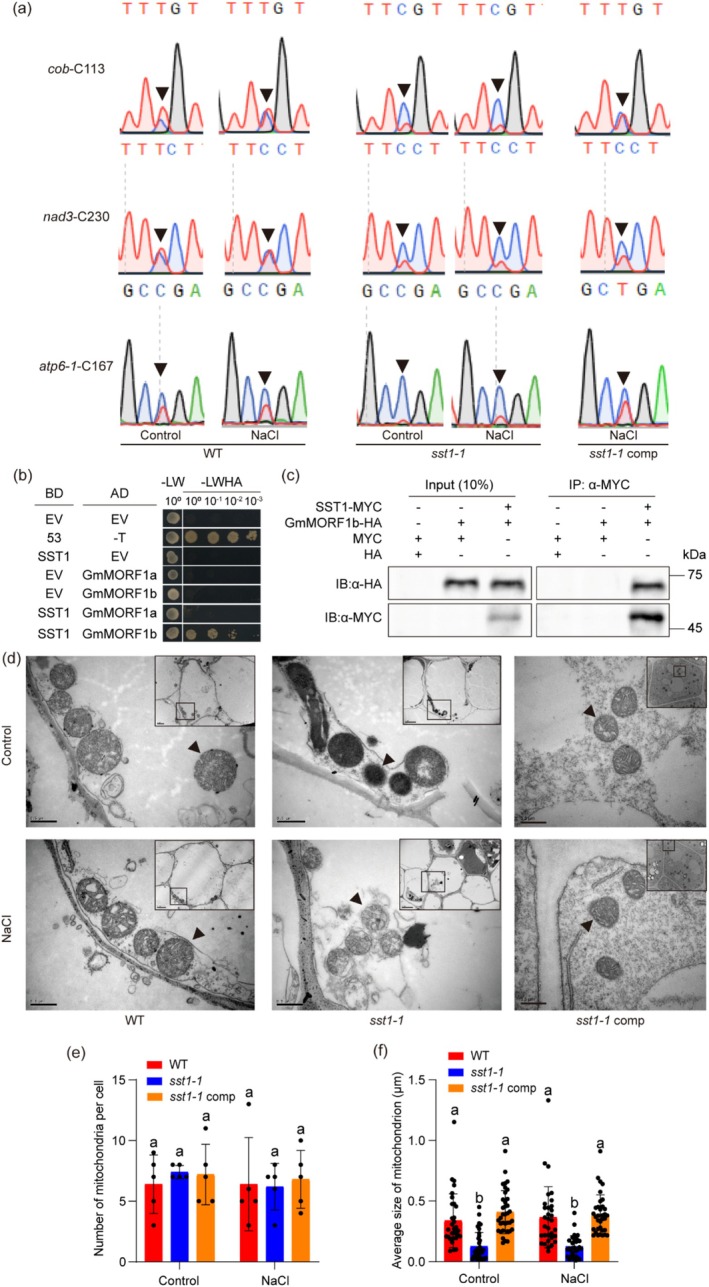
RNA editing of *cob*, *nad3* and *atp6‐1* of WT, *sst1‐1* mutant and *sst1‐1* comp and mitochondrial morphology of WT and *sst1‐1* mutant. (a) *SST1* affects the RNA editing efficiency of the mitochondrial genes *cob*, *nad3* and *atp6‐1*. The black arrows point to the editing site. (b) Y2H analysis to detect the interactions between SST1 and GmMORF1a or GmMORF1b. Different combining vectors of pGBKT7‐ and pGADT7‐ were cultured in selective media lacking Leu and Trp (SD/‐2) and subsequently in media lacking Ade, His, Leu and Trp (SD/‐4) to test protein–protein interactions. (c) CoIP assay of interaction between SST1 and GmMORF1b. SST1‐MYC and GmMORF1b‐HA were transiently expressed under the control of the 35 S promoter in *N. benthamiana* leaves. SST1‐MYC immunoprecipitated from total protein extracts. (d) The transmission electron microscope images of mitochondria in WT, *sst1‐1* and *sst1‐1* comp. Bars = 0.5 μm. The black arrows point to the mitochondria. The upper right panel showed the complete cell. (e, f) Number of mitochondria per cell and average size of mitochondrion of the WT, *sst1‐1* and *sst1‐1* comp. Different letters indicate statistically significant differences at *p* < 0.05 based on a two‐way ANOVA test. *n* = 5.

The PLS‐class PPR proteins involved in organelle RNA editing recognise the 5′ upstream sequence of an editable cytidine (C), with the last C‐terminal PPR motif positioned at nucleotide position 4 (four nucleotides upstream) with respect to the editing site (Barkan et al. 2012; Takenaka et al. 2013; Yagi et al. 2013; Okuda et al. 2014; Kindgren et al. 2015). This conserved alignment rule for RNA‐edited PPRs allows the computational prediction of target RNA editing sites via amino acid sequences of PLS‐class PPR proteins (Kobayashi et al. 2019). To investigate the role of SST1 in mitochondrial RNA editing, we first identified the potential target genes of SST1 by analysing the predicted targets for an *Arabidopsis* PPR protein (AT5G50390) (Kobayashi et al. 2019), which shares the highest similarity with SST1 (Figure S6b), and the editing status of these predicted targets was then analysed in the *sst1‐1* mutant via direct sequencing. Among the 26 transcripts obtained and sequenced, three mitochondrial genes exhibited abnormal editing. In the WT, there was a partial conversion of the C nucleotide to T in the cDNA sequences of C113 of *cob* (cytochrome b) (Liberatore et al. 2016), C230 of *nad3* (NADH dehydrogenase subunit 3) (Yuan and Liu 2012) and C167 of *atp6‐1* (ATP synthase F0 subunit 6) (Arimura et al. 2020). In contrast, in the *sst1‐1* mutant, these editing sites retained the original unedited C nucleotide (Figure 5a). To investigate whether SST1 is involved in the editing process, we overexpressed *SST1* ^Hap3/HapC^ in the *sst1‐1* mutant and tested the editing status of these three genes. Overexpression of *SST1* ^Hap3/HapC^ restored the editing of these three genes to wild‐type level (Figure 5a). In addition, we overexpressed *SST1* ^Hap2/HapT^ and *SST1* ^Hap3/HapC^ in Changnong 17 (the haplotype is *SST1* ^Hap2/HapT^). In the *SST1* ^Hap3/HapC^ transgenic hairy roots, the editing of *cob*, *nad3* and *atp6‐1* at the tested sites showed a similar pattern as W82 with partial C to T, while in the *SST1* ^Hap2/HapT^ transgenic hairy roots, the editing of these three genes remained identical to those in Changnong 17 (Figure S17). These results indicate that SST1 is required for editing at these RNA editing sites in mitochondrial genes.

To investigate how SST1 affects RNA editing of mitochondrial genes, we examined whether SST1 interacts with the known proteins that are involved in this process. MORF1 is one of the proteins required for multiple editing events in plant mitochondria (Takenaka et al. 2012; Bentolila et al. 2013). We then conducted yeast two‐hybrid (Y2H) and co‐immunoprecipitation (CoIP) assays to test whether SST1 interacts with MORF1. As shown in Figure 5b, SST1 demonstrated a strong interaction with GmMORF1b, but not with GmMORF1a (Figure 5b). Furthermore, CoIP confirmed the in vivo interaction between SST1 and MORF1b (Figure 5c). These results indicate that SST1 affects RNA editing of *cob*, *nad3*, and *atp6‐1* through its interaction with GmMORF1b.

Mitochondria, the primary energy source of plant cells, are highly dynamic organelles that maintain cellular energy homeostasis. The energetic states of eukaryotic cells are closely associated with mitochondrial morphology and inner structure (Garcia et al. 2019). Because SST1 regulates the RNA editing of mitochondrial genes *cob*, *nad3* and *atp6‐1*, we investigated whether SST1 affects mitochondrial morphology and structure of root tips by transmission electron microscopy under normal and salt stress conditions (Figure S18). There was no significant difference in the number of mitochondria between the *sst1‐1* and WT plants under either condition (Figure 5e). In sharp contrast, the size, morphology, and inner structure of mitochondria in the *sst1‐1* mutant root cells were changed substantially regardless of growth conditions (Figure 5d,f). The average size of the mitochondria in the mutant cells was much smaller than that in the WT cells (Figure 5d,f). Specifically, swollen cristae with vesicle‐like structures and reduced intermembrane content were observed in the mitochondria of *sst1‐1* mutant root cells (Figure 5d). In addition, we investigated the morphology of mitochondria in the *sst1‐1* complementary line using hairy root transformation technology. The results showed that the morphology and internal structure of root tip mitochondria in complementary soybean lines were restored to WT levels under both control and salt stress conditions (Figure 5d–f, Figure S19a).

To validate the relationship between mitochondrial functions and salt tolerance. We first measured the activity of complex I‐complex IV in the electron transport chain under control and salt stress conditions in WT and *sst1‐1* mutant. In WT, the activity of complexes I, III and IV was significantly decreased by stress, and the activity of complex IV was significantly decreased in the *sst1‐1* mutant under both control and salt stress conditions (Figure S19b–e). We then tested the ATP synthesis ability of WT and *sst1‐1* mutants under control and salt stress conditions. The ATP synthesis ability was decreased in WT under salt stress conditions compared with the control condition. For the *sst1‐1* mutant, the ATP synthesis ability was decreased in both control and salt stress conditions compared with WT (Figure S19f). These results indicate that salt inhibits mitochondrial functions and SST1 plays a critical role in these processes. Taken together, these results indicate that SST1 is essential for RNA editing of mitochondrial ATP synthesis genes and normal mitochondrial function and morphology.

### 2.6 Gene Networks Are Differentially Regulated by *SST1* Under Salt Stress

**FIGURE 6:**
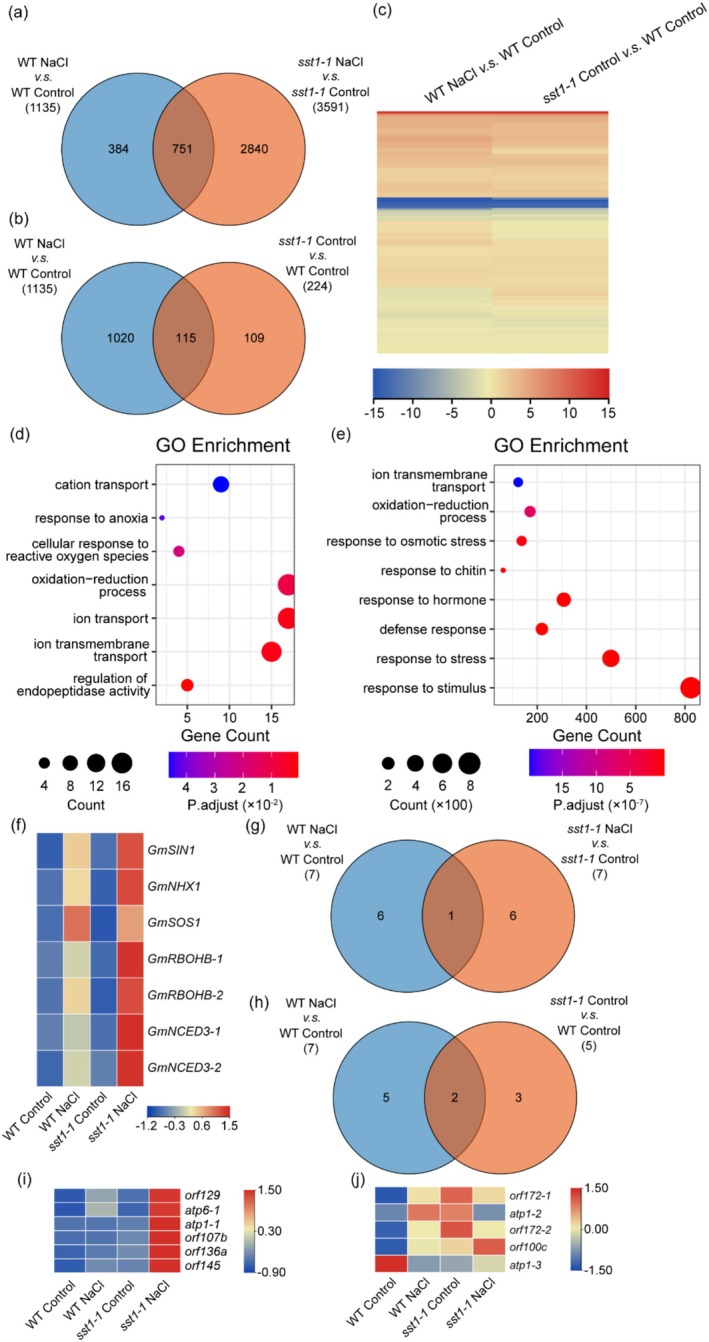
Transcriptome analysis of *sst1‐1* mutant and WT. (a, b) Venn diagrams showing the extent of overlap between nuclear genome differentially expressed genes (DEGs) in *sst1‐1* mutant and WT under control (0 mM NaCl) or NaCl (100 mM NaCl) treatments. (c) Hierarchical clustering analysis of common nuclear genome DEGs from control‐treated WT vs. NaCl‐treated WT and *sst1‐1* mutant vs. WT comparisons. Numerical values in the blue‐to‐red gradient bar represent Log_2_(fold‐change) relative to the control sample. (d) Gene ontology (GO) terms statistically enriched among nuclear genome DEGs (224 genes) in control‐treated *sst1‐1* mutant vs. control‐treated WT RNA‐seq comparison. (e) GO terms statistically enriched among nuclear genome‐specific expressed DEGs (2840 genes) in NaCl‐treated *sst1‐1* mutant vs. control‐treated *sst1‐1* mutant RNA‐seq comparison. (f) Heatmap of gene expression of salt stress‐related genes in *sst1‐1* mutant vs. WT. Numerical values in the blue‐to‐red gradient bar represent Log_2_(fold‐change) relative to the control sample. (g, h) Venn diagrams showing the extent of overlap between mitochondrial genome DEGs in *sst1‐1* mutant and WT under control (0 mM NaCl) or NaCl (100 mM NaCl) treatments. (i) Heatmap of mitochondrial genome‐specific expressed DEGs (6 genes) in NaCl‐treated *sst1‐1* mutant vs. control‐treated *sst1‐1* mutant. Numerical values in the blue‐to‐red gradient bar represent Log_2_(fold‐change) relative to the control sample. (j) Heatmap of mitochondrial genome DEGs (5 genes) in control‐treated *sst1‐1* mutant vs. control‐treated WT. Numerical values in the blue‐to‐red gradient bar represent log_2_(fold‐change) relative to the control sample.

To further elucidate the molecular mechanism of *SST1* under salt stress, we performed transcriptome analysis of the nucleus and mitochondria using ribosome‐depleted RNA sequencing (RNA‐seq). We identified a total of 1135 nuclear genome differentially expressed genes (DEGs; 662 up‐regulated and 473 down‐regulated) in the comparison between control‐ and NaCl‐treated WT. Interestingly, NaCl treatment caused significant transcriptomic changes in the *sst1‐1* mutant compared with WT, with 3591 nuclear genome DEGs (2050 up‐regulated and 1541 down‐regulated) (Figure 6a, Table S3). Additionally, we identified 224 (144 up‐regulated and 80 down‐regulated) DEGs between control‐treated WT and control‐treated *sst1‐1* mutant (Figure 6b, Table S4). These results suggest that the *sst1‐1* mutant is more sensitive to NaCl treatment than WT in terms of transcriptomic responses.

To better understand the relationship between NaCl treatment‐induced DEGs (DEGs in NaCl‐treated WT vs. DEGs in control‐treated WT) and *sst1‐1* mutant‐induced DEGs (DEGs in control‐treated *sst1‐1* mutant vs. DEGs in control‐treated WT), we analysed the expression patterns of 115 DEGs using hierarchical clustering (Figure 6c). The results showed that almost all DEGs had identical expression patterns. Gene ontology (GO) analysis further revealed that most of the genes regulated by *SST1* are involved in ion transport, oxidation–reduction processes and anoxia response (Figure 6d, Table S5). Similarly, we found that most of the genes in the *sst1‐1* mutant specifically were involved in stimulus response, stress response, hormone response, and osmotic stress response under NaCl treatment (Figure 6e, Table S6). These results further support that *SST1* is involved in a variety of responses to salt stress. Furthermore, we examined the expression of salt stress‐related genes in the transcriptome. Our analysis revealed that the expression levels of these salt stress‐related genes were consistent with the qPCR results, indicating that the absence of *SST1* indeed affects the expression changes of these genes (Figures 3h, 6f, Table S7).

At the same time, we also analysed the transcriptome of the mitochondrial genome. We identified 7 mitochondrial genome DEGs (4 up‐regulated and 3 down‐regulated) in the comparison between control‐ and NaCl‐treated WT. NaCl treatment caused a similar change in the transcriptome in the *sst1‐1* mutant compared with WT, with 7 mitochondrial genome DEGs (6 up‐regulated and 1 down‐regulated) (Figure 6g, Table S8). We also identified 5 (4 up‐regulated and 1 down‐regulated) DEGs between control‐treated WT and the *sst1‐1* mutant (Figure 6h, Table S9). These results suggest that *sst1‐1* mutant is more sensitive to NaCl treatment than WT in terms of transcriptomic changes. Overall, the sensitivity to NaCl treatment was similar between the *sst1‐1* mutant and WT.

To further analyse the effect of *SST1* on mitochondrial gene transcription specifically, we identified 6 mitochondrial genes that were differentially expressed in the *sst1‐1* mutant. These genes, including mitochondrial complex V (*atp6‐1* and *atp1‐1*), were significantly up‐regulated in the *sst1‐1* mutant under NaCl treatment (Figure 6i, Table S10). Similarly, we also identified the transcriptional effect of *SST1* on mitochondrial genes under control treatment. We found that 5 genes, including mitochondrial complex V (*atp1‐2* and *atp1‐3*), were differentially expressed in the *sst1‐1* mutant (Figure 6j, Table S10). These data further indicate that *SST1* affects the transcription level of some mitochondrial genes (such as mitochondrial complex V) in the early stages of control and NaCl treatment.

### 2.7 The *SST1* Gene Originates from Wild Soybean and Is Mutated in Domesticated Varieties

**FIGURE 7:**
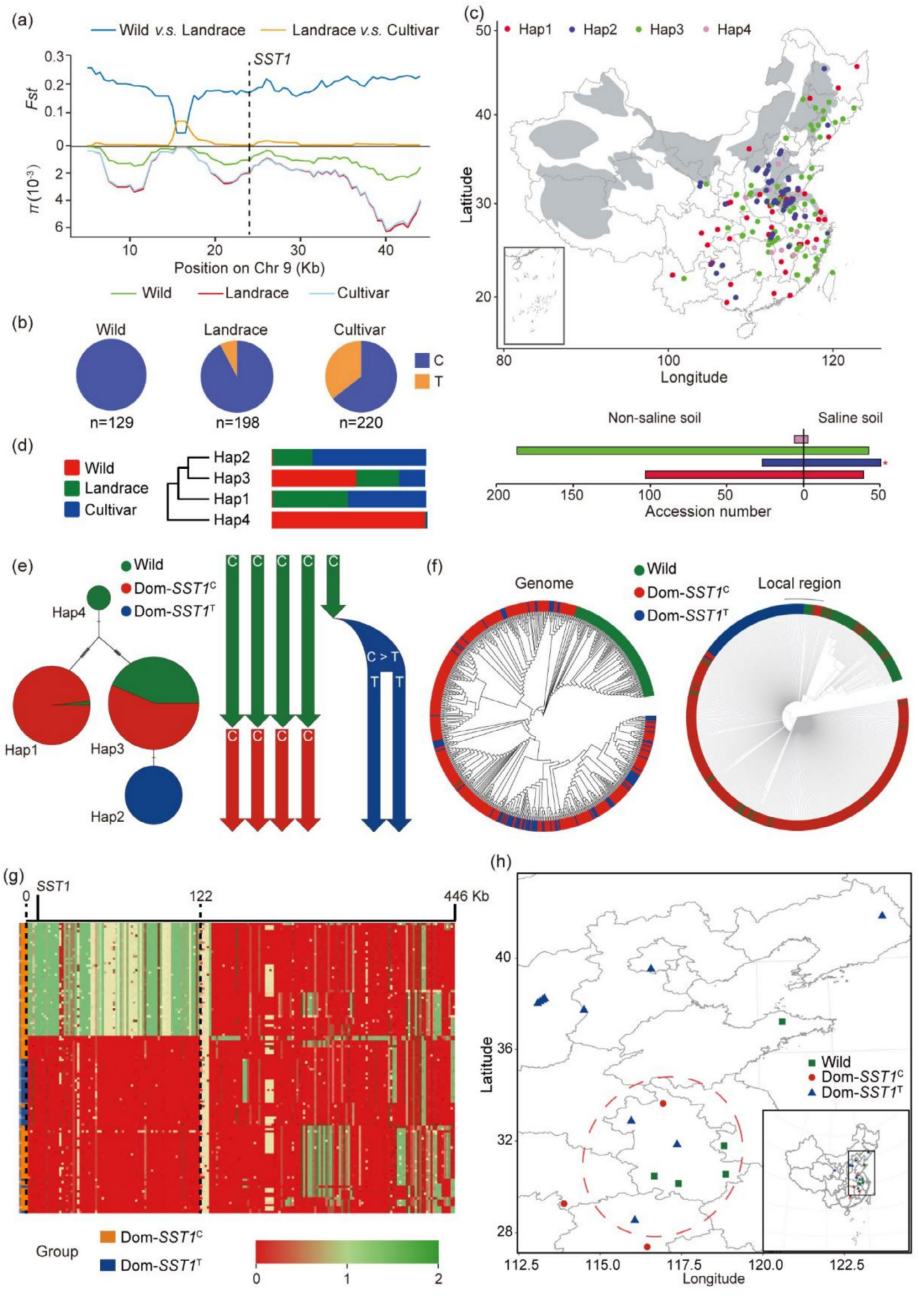
Diversity analysis, geographic distribution and dynamic interspecific transfer of *SST1* alleles in soybean. (a) *F*st and nucleotide diversity between wild, landrace and cultivar accessions across the 44.2 kb region containing *SST1*. The vertical dotted black line indicates the physical position of *SST1*. (b) Proportions of *SST1* alleles and their co‐occurrence within each of the three subgroups. (c) The upper panel shows the geographical distribution of four haplotypes in *SST1*. The regions of saline‐affected soil are presented in grey on the map. The lower panel shows the proportion of four haplotypes of *SST1* in saline and non‐saline soil. Student's *t*‐test was used to generate *p* values. (d) Phylogenetic tree of the four haplotypes. The right panel shows the proportion of each haplotype in the three subgroups. (e) Haplotype network and domestication model of *SST1*. Left panel shows the haplotype network. Circle size is proportional to the number of samples for three subgroups. Right panel shows the domestication model of *SST1*. (f) A global (left panel) and a local (right panel) phylogenetic tree constructed from 559 wild, landrace and cultivar accessions using genome‐wide SNPs and SNPs spanning the 117.19 kb region containing *SST1*, respectively. Labels indicate three clusters of accessions. (g) Pairwise nucleotide distance analyses of cultivar accession with two domestication accessions (Dom‐*SST1* ^C^ and Dom‐*SST1* ^T^) with its closest wild accession (GDW082) across the 446‐Kb region. (h) Interspecific allele transfer. Geographic origins of Dom‐*SST1* ^T^ accessions and their closest wild accessions are shown (accessions marked by grey curve in the local tree). Dotted circles depict the geographic regions in China where the interspecific allele transfer might occur. Latitude and longitude are shown.

To investigate the evolution of the *SST1* gene in soybean, we analysed its natural variation and population genetic parameters. This analysis utilised genomic sequences from 559 soybean germplasms, including wild, landrace and cultivar accessions. We assessed *Fst* and nucleotide diversity (π) across a 44.2 kb genomic region surrounding *SST1*, identifying robust evidence of selection within this region (Figure 7a). These findings suggest that the *SST1* gene has been under strong natural selection throughout its evolutionary history. Interestingly, analysis of the polymorphism at SNP24932, which induces early termination of *SST1* transcripts, revealed that *SST1* is subject to strong purifying selection in wild soybean. The new allele (T), which emerged in landraces, was further selected in elite cultivars, indicating that *SST1* likely plays a crucial role in the adaptation of wild soybean and that the C‐to‐T mutation resulted in a loss of *SST1* function, increasing salt stress tolerance, and was then preferentially selected during soybean improvement (Figure 7b). To explore the correlation between the geographic distribution of the *SST1* haplotypes and saline soil environments (Liu and Wang 2021), we mapped all germplasms to their associated soil types. We found that varieties carrying Hap2 were significantly more prevalent in saline soils than other haplotypes (Figure 7c).

To elucidate the potential origins of the *SST1* haplotypes, we conducted phylogenetic analysis and haplotype network analysis on the four haplotypes. The phylogenetic tree constructed with these haplotypes revealed that Hap4 was different from the other three haplotypes, with Hap2 and Hap3 showing close relationships (Figure 7d). Moreover, to explore the domestication trajectory of the SNP24932 locus in soybean, we categorised the 559 germplasms into wild accessions and domestication accessions (landrace and cultivar), with domestication accessions further classified into C and T variants on the basis of the SNP24392 mutation, referred to as Dom‐*SST1* ^C^ and Dom‐*SST1* ^T^. Haplotype network analysis revealed that Dom‐*SST1* ^C^ in Hap1 and Hap3 originated from Hap4 in wild soybean, whereas Dom‐*SST1* ^T^ evolved from Dom‐*SST1* ^C^ in Hap3 (Figure 7e). We also compared the phylogenetic relationships of the 559 accessions via SNPs from genome‐wide and local regions (44.2 kb) surrounding the *SST1* gene. All domestication accessions (Dom‐*SST1* ^C^ and Dom‐*SST1* ^T^) clustered together and were separated from the wild accessions (Figure 7f). However, in the local tree, the Dom‐*SST1* ^T^ accessions closely aligned with the wild accessions (Figure 7f), suggesting introgression of the *SST1* alleles between the wild and domesticated forms. To assess this hypothesis, we estimated and plotted pairwise genetic distances between each of the 358 domesticated accessions (distributed in Dom‐*SST1* ^C^ and Dom‐*SST1* ^T^) and a representative wild cluster (GDW082) across the 446 kb region to identify potential genomic transfers of the *SST1* region. This analysis revealed two distinct patterns of sequence variability within the region among the groups. Approximately 52% (52.63%) of the Dom‐*SST1* ^C^ accessions presented no introgressed segments in a 122 kb region around *SST1*; conversely, all the Dom‐*SST1* ^T^ and the remaining Dom‐*SST1* ^C^ accessions presented evidence of chromosomal fragment infiltration from wild sources in this region (Figure 7g). These findings support the hypothesis that Dom‐*SST1* ^T^ in these domestication accessions likely originated from a postdomestication allele introgression event. To ascertain the geographical origin of these accessions, we mapped the locations of the closest related Dom‐*SST1* ^T^ and wild accessions identified on the periphery of the local tree. The data indicated that the most closely related Dom‐*SST1* ^*T*^ and wild accessions are located in the Huanghuai region of China (Lu et al. 2020), encompassing the lower reaches of the Yellow River (Huang He) and the Huai River, including parts of provinces such as Henan, Shandong, Anhui and Jiangsu, particularly in Anhui Province (Figure 7h), suggesting that interspecific hybridisation between wild and domesticated soybeans most likely occurred in these areas.

### 2.8 The *SST1* and *GmCHX1* Genes Synergistically Regulate Plant Salt Tolerance

**FIGURE 8:**
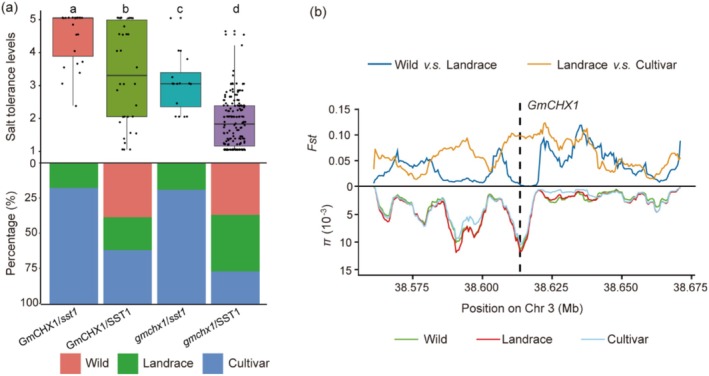
Genetic relationship between *GmCHX1* and *SST1* in salt tolerance and diversity analysis of the *GmCHX1* gene. (a) The different genetic relationships between *GmCHX1* and *SST1* were associated with salt tolerance levels and percentages in the three subgroups. The upper panel shows the distributions of salt tolerance levels for each genetic relationship. Different letters indicate statistically significant differences at *p* < 0.05 based on the Kolmogorov–Smirnov test. The lower panel shows the percentage distribution of four genetic relationships in wild populations, landraces and cultivars. (*n* = 23 for *GmCHX1*/*sst1*; *n* = 34 for *GmCHX1/SST1*; *n* = 16 for *gmchx1*/*sst1*; *n* = 164 for *gmchx1*/*SST1*). (b) *F*st and nucleotide diversity between wild, landrace and cultivar accessions across the 260 kb region containing *GmCHX1*. The vertical dotted black line indicates the physical position of *GmCHX1*.

In our GWAS analysis, *SST1* was simultaneously identified with a stronger locus on chromosome 3 (Figure 1e), which positively regulates salt tolerance through *GmCHX1* in soybean (Guan et al. 2014; Qi et al. 2014). This finding led us to explore the genetic relationship between *GmCHX1* and *SST1* in soybean salt tolerance. We compared the relationships between the genotypes and salt tolerance of 240 soybean accessions. As the new allele (T) in SNP24932 causes SST1 to produce a truncated protein, SST1 was divided into two categories: one with a new allele, named *sst1*, and another type named *SST1*. Based on the genotypic analysis of *GmCHX1* by Qi et al. (2014), the *GmCHX1* genotype with salt tolerance was named *GmCHX1*, and the *GmCHX1* loss‐of‐function genotype as *gmchx1*. The results revealed that *GmCHX1*/*sst1* conferred strong salt tolerance to soybean, whereas *gmchx1*/*SST1* reduced the salt tolerance of soybean, which is consistent with their positive and negative effects on plant salt tolerance, respectively (Figure 8a). Next, we compared the effects of the *GmCHX1*/*SST1* and *gmchx1*/*sst1* on plant salt tolerance. Both the *GmCHX1*/*SST1* and *gmchx1*/*sst1* conferred moderate salt tolerance to soybean, but *GmCHX1*/*SST1* presented greater salt tolerance than *gmchx1*/*sst1* did (Figure 8a). Taken together, these results suggest that *SST1* and *GmCHX1* have additive effects on salt tolerance, with *GmCHX1* exerting a stronger effect than *SST1* on soybean salt tolerance.

Interestingly, among the four genotype combinations, mutations in *SST1* are more prevalent in cultivars, less common in landraces and absent in wild species. In contrast, mutations in *GmCHX1* exhibited no obvious distinct pattern across these germplasms. These results suggest that *SST1* is undergoing domestication, whereas *GmCHX1* is unlikely to have undergone domestication. To investigate whether *GmCHX1* has undergone artificial domestication, we performed *F*st and nucleotide diversity analyses. The results revealed no obvious traces of domestication at *GmCHX1*, at least on the basis of our data (Figure 8b).

## 3 Discussion

Soybean is a moderately salt‐tolerant species (Munns and Tester 2008; Do et al. 2016). Salt stress affects every stage of plant growth and development, leading to poor germination, stunted growth, leaf and plant death and a low yield of soybean (Singleton and Bohlool 1984; Ashraf and Wu 1994; Chang et al. 1994; Xu et al. 2017; Zhang et al. 2018). Although there have been breakthroughs in understanding the molecular mechanisms of salt tolerance in soybeans, the evolution of salt tolerance genes during domestication and genetic breeding remains unclear. In this study, we drew on the phenotyping protocols of previous research (Tuyen et al. 2010) and further simplified them to develop a new reliable system (SILS) for evaluating salt tolerance of soybean seedlings. Using this system, we identified two main loci, including a known *GmCHX1* gene and one new locus for salt tolerance through GWAS, and importantly, we functionally characterised *SST1* as a new salt tolerance‐related and adaptive domestication‐related gene. We also revealed the additive effects of *SST1* and *GmCHX1* on the salt tolerance of soybean.

Soybeans are annual crops cultivated in temperate, subtropical and tropical regions worldwide. The germination and growth of seedlings are substantially affected when soybean is planted in the saline‐alkali soil areas. Owing to the complexity of the salt tolerance of soybean, many treatments at different stages and scoring systems (germination and emergence rates, ion content, biomass, leaf injury, etc.) for the salt tolerance of seed germination, emergence and seedlings have been used for the evaluation of salt‐tolerant germplasms and the identification of salt tolerance genes (Ledesma et al. 2016; Zhang et al. 2019; Jin et al. 2021). In this study, we evaluated the salt tolerance of soybean during seed germination, emergence and seedling growth. Most importantly, we identified two main loci using GWAS, including a known salt‐tolerant gene, *GmCHX1*, and one new genetic locus for salt tolerance in the natural population used (Figure 1e). Further functional study of one locus on chromosome 9 led to the identification of *SST1* as a new salt tolerance gene in soybean. Intriguingly, comparative GWAS analysis revealed a significant salt tolerance QTL on chromosome 19 when using the ZH13 reference genome, while no corresponding signal was detected in the W82.a2 assembly. This discrepancy likely stems from assembly errors in the W82.a2 genome that compromised accurate gene localization. The identified QTL region in ZH13 warrants further investigation to characterise the underlying candidate genes governing salt tolerance.

Soybean salt tolerance involves various biological processes, including osmotic stress, ion toxicity, and disruption of ROS homeostasis (Jones 2000; Mittler et al. 2022). The results from GWASs have led to the identification of many loci and genetic variants on different chromosomes associated with salt tolerance at the germination and seedling stages. However, very few genes (such as *GmCDF1*) and genetic variants have been shown to play a role in the salt tolerance of soybean (Zhang et al. 2019). Interestingly, the genes identified from genetic populations are mainly genes related to ion or cation transporters, such as *GmCHX1* and *GmCDF1* (Guan et al. 2014; Qi et al. 2014; Zhang et al. 2019). In this work, we identified *SST1* as a salt tolerance gene through a GWAS. We showed that SNP24932 in the coding region of the *SST1* gene forms a premature stop codon, resulting in loss of function of the gene and increased salt tolerance in plants (Figures 2 and 3). The negative regulatory role of the *SST1* gene in salt tolerance was validated via overexpression and CRISPR/Cas9 analyses (Figure 3; Figure S3). Physiological results revealed that a loss‐of‐function mutation in SST1 significantly reduced the ratio of Na^+^/K^+^ in leaves but increased the Na^+^/K^+^ ratio in roots (Figure 3d,e), suggesting that SST1 promotes root‐to‐shoot transport and accumulation of Na^+^, thereby negatively regulating the salt tolerance of plants. We also observed that the loss‐of‐function mutant *sst1* accumulated relatively high levels of H_2_O_2_ in roots under salt stress (Figure 3f,g), indicating that SST1 modulates the salt tolerance of plants by affecting ROS homeostasis. The findings that the SST1 mutation affects transcriptomic responses of soybean plants to salt stress (Figure 6a–f), including the expression of salt stress‐related genes, such as *GmNHX1*, *GmSOS1*, *GmSIN1*, *GmNCED3s* and *GmRbohBs* (Figure 3h), indicate that SST1 modulates plant salt tolerance by regulating various biological processes, in particular ion accumulation and ABA and ROS homeostasis.

*SST1* encodes a PPR protein and is localised in mitochondria (Figure 4d; Figure S16). PPR family proteins exert their functions by regulating RNA editing in organelles, including mitochondria. C‐to‐U RNA editing represents a widespread post‐transcriptional modification in plant chloroplasts and mitochondria, mediated by site‐specific cytidine deamination (Blanc et al. 1995). PPR proteins, particularly those containing a DYW domain, serve as essential factors for site‐specific C‐to‐U RNA editing in plant organelles. The DYW domain harbours a conserved zinc‐binding motif (HxE(x)nCxxC) that exhibits structural similarity to cytidine deaminases, with extensive experimental evidence supporting its direct catalytic role in the deamination reaction (Bhattacharya et al. 1994). Mutagenesis studies of the DYW domain's deaminase motif (HxE(x)nCxxC) in Arabidopsis proteins DYW1, OTP84 and CREF7 demonstrate that zinc‐coordinating residues are essential for structural integrity, while the conserved glutamate mediates catalytic proton transfer, with mutations in these critical residues completely abolishing C‐to‐U editing activity (Boussardon et al. 2014; Hayes et al. 2015). Structural analyses have unequivocally established that the DYW domain adopts a canonical cytidine deaminase fold containing dual zinc centers—a catalytic zinc atom that directly mediates deamination chemistry and a structural zinc atom that maintains domain architecture, with the characteristic DYW motif orchestrating precise coordination geometry of the catalytic zinc through its zinc‐stabilised conformation, thereby mechanistically controlling the RNA editing reaction (Takenaka et al. 2021). The presence of a DYW domain in SST1, along with impaired C‐to‐U RNA editing at specific mitochondrial transcripts (*cob*, *nad3* and *atp6‐1*) in *sst1* mutants, and its interaction with GmMORF1b, suggests that SST1 acts as an RNA editing factor, similar to its characterised homologues.

Intriguingly, our results revealed that SST1 is responsible for the RNA editing of *cob*, *nad3* and *atp6‐1* RNA (Figure 5a). The *cob* gene encodes a subunit of the mitochondrial electron transfer chain complex III, in which it participates in the electron transport chain process. Salt stress leads to excessive reduction of the mitochondrial electron transfer chain (mtETC), resulting in electron leakage to O_2_ and subsequent production of ROS, especially H_2_O_2_ (Liberatore et al. 2016). In plants, mitochondria can bypass the oxidative phosphorylation pathway and transport protons without producing ATP, reducing superoxide production by mtETC via alternative NAD(P)H dehydrogenases (NDs) and alternative oxidases (AOXs) (Vanlerberghe 2013). The *nad3* gene encodes a part of the NADH dehydrogenase subunit, which plays a crucial role in the cellular respiration process. Previous studies have shown that the *Arabidopsis* PPR protein slow growth 1 (SLG1) regulates the plant response to ABA and tolerance to drought stress through RNA editing of *nad3*. Compared with the wild‐type, the *slg1* mutant accumulates higher levels of H_2_O_2_ in guard cells (Yuan and Liu 2012). Given the involvement of SST1 in the RNA editing of both the *cob* and *nad3* genes, *SST1* modulates mitochondrial functions, including the electron transport chain and the cellular respiration process, to regulate the ROS homeostasis and subsequent salt tolerance of soybean plants. Furthermore, the *atp6* gene encodes subunit a of ATP synthase and is essential for generating ATP to maintain plant survival. In *Arabidopsis*, a single mutation in either the *atp6‐1* or *atp6‐2* gene results in normal plant growth, whereas the loss of function of both the *atp6‐1* and *atp6‐2* genes is lethal, suggesting functional redundancy of these two genes in terms of plant survival (Arimura et al. 2020). Here, we showed that a mutation in *SST1* affects the transcription level of some mitochondrial genes (such as mitochondrial complex V) (Figure 6g–j) and specifically reduces the RNA editing of *atp6‐1* (Figure 5a), suggesting that SST1 also modulates plant salt tolerance through ATP production. The presence of functional redundancy may explain the normal growth of *sst1* mutant plants compared with that of wild‐type plants under normal conditions. Unexpectedly, our results revealed that decreasing RNA editing of *cob*, *nad3* and *atp6‐1* in the *sst1* mutant seems to increase the salt tolerance of the mutant plants. Given that SST1 specifically interacts with MORF1b (Figure 5b,c), a member of MORF proteins that interact with PPR proteins to recognise the editing site, we speculate that SST1 protein affects MORF1b's ability to recognise the editing site, resulting in a reduction in *cob*, *nad3* and *atp6‐1* RNA editing and changes in mitochondrial morphology to trigger the signalling pathway that promotes salt tolerance in soybean. This hypothesis was supported by the finding that the loss of function of *SST1* dramatically affected mitochondrial crista structure, size, and morphology regardless of normal or salt stress conditions (Figure 5d–f). Previous studies have shown that ATP synthase oligomerisation affects crista architecture and mitochondrial morphology (Habersetzer et al. 2013). The loss of function of SST1 may increase salt tolerance by affecting ATP synthase and subsequently mitochondrial structure and morphology. However, it is unclear whether alterations in mitochondrial morphology promote the salt tolerance of soybean plants by increasing mtROS generation.

Plant cells possess both anterograde (nucleus‐to‐organelle) and retrograde (organelle‐to‐nucleus) signalling pathways (Huang et al. 2016). Among PPR proteins, retrograde signalling appears to be more prevalent than anterograde signalling (Huang et al. 2016). For instance, SLG1, a mitochondrion‐localised PPR protein in Arabidopsis, is required for RNA editing at specific sites in the mitochondrial *nad3* transcript. *slg1* mutants disrupt this editing process, leading to the upregulation of alternative respiratory pathway genes, which may compensate for mitochondrial dysfunction and improve stress tolerance (Yuan and Liu 2012). Similarly, mutations in *SLO2*, another mitochondrial PPR protein in Arabidopsis, enhance salt stress tolerance. Transcriptome analysis revealed that the *slo2* mutant alters the expression of multiple stress‐responsive genes, suggesting that retrograde signalling may contribute to its increased salt tolerance compared to the wild type (Zhu et al. 2014). Given these findings, SST1 might regulate stress adaptation through a similar retrograde signalling mechanism, thereby enhancing salt tolerance in soybean.

Notably, a significant difference in nucleotide polymorphisms was detected within the genomic region encompassing *SST1* between wild and domesticated soybean accessions (Figure 7a). Specifically, wild soybeans presented significantly lower nucleotide diversity than their domesticated counterparts did (Figure 7a), a pattern that deviates from typical domestication scenarios where domesticated varieties often present reduced genetic diversity (Huang et al. 2022). This anomaly suggests that the *SST1* region may harbour crucial variations for adaptation to environments, which underwent weak selection pressure during soybean domestication, thereby increasing genetic diversity at this locus. The lower degree of polymorphism in wild soybeans at the *SST1* locus indicates the conservation of essential genes related to survival, suggesting the presence of potential undiscovered genes pivotal for environmental adaptation. Overall, loss‐of‐function mutations in *SST1* ^Hap2/HapT^ were strongly selected and rapidly fixed in cultivars, making cultivars more adaptable to saline‐alkali soils. The region around *SST1* underwent selective and adaptive domestication. In addition, the geographic distribution of different alleles of *SST1* underscores the adaptive expansion of *SST1* ^Hap2^ in saline environments during soybean cultivation (Figure 7c). These findings align with previous studies showing that advantageous allelic variations in salt tolerance genes, such as *GmCHX1* and *E2*, are naturally selected in high‐salinity regions (Guan et al. 2014; Dong et al. 2022). The local genomic region containing *SST1* shows closer genetic affinity between the Dom‐*SST1* ^T^ variants and wild soybeans. Based on this, we propose a model of *SST1* involvement in soybean domestication. We believe that chromosome introgression from wild soybean to domesticated soybean is the result of Dom‐*SST1* ^T^ occurrence (Figure 7e). Reports of natural gene flow between wild and cultivated soybeans are widespread (Wang et al. 2019), paralleling similar introgression events documented in rice and maize (Zhao et al. 2010; Hufford et al. 2013; Wang et al. 2017). Thus, this study provides compelling evidence for the role of *SST1* in soybean domestication and highlights the importance of genetic introgression in increasing crop resilience to environmental stresses. Based on this, we believe that *SST1* is an effective gene for breeding new salt‐tolerant soybean varieties.

Interestingly, in our GWAS, the identification of the SST1 mutation in soybean salt tolerance consistently coincides with the detection of *GmCHX1*, a known salt tolerance gene in soybean, suggesting a relationship between the two genes in the regulation of soybean salt tolerance. *GmCHX1* had a stronger effect on salt tolerance than did *SST1* (Figure 1e), indicating that *GmCHX1* is a major salt tolerance locus in soybeans. However, the introduction of superior allelic variations of *SST1* into the genetic background with the tolerant *GmCHX1* allele significantly increases soybean salt tolerance (Figure 8a), suggesting that *GmCHX1* and *SST1* synergistically regulate soybean salt tolerance. In summary, our results not only identified a new salt tolerance gene and a superior variant but also demonstrated its additive effect with GmCHX1 in salt tolerance in soybean. This provides a new avenue for elucidating the molecular network of salt tolerance in plants and breeding new salt‐tolerant soybean germplasms through superior gene aggregation and pyramiding. This will provide an option for growing soybean in saline soils to increase soybean production.

## 4 Materials and Methods

### 4.1 Plant Growth and Assessment of Salt Tolerance of Soybean

Soybean ( *G. max* and *G. soja* ) plants were grown in a greenhouse (16 h: 8 h, light: dark, 25°C, 50% relative humidity) in Wuhan (30°28′ N, 114°21′ E) in vermiculite. Soybean seeds were planted in pots (10 cm by 10 cm) containing autoclaved vermiculite. Three to five biological replicates were designed for each accession. The seedlings were then subjected to alternating irrigations with water and Broughton and Dilworth normal nitrogen fertiliser solution containing nitrate (7.88 mM), KH_2_PO_4_ (0.068 g/L), MgSO_4_·7H_2_O (0.0616 g/L), K_2_SO_4_ (0.0435 g/L), (NH_4_)_2_SO_4_ (0.3884 g/L), MnSO_4_·H_2_O (0.1690 mg/L), H_2_BO_3_ (0.1237 mg/L), ZnSO_4_·7H_2_O (0.1438 mg/L), CuSO_4_·5H_2_O (0.0499 mg/L), CoSO_4_·7H_2_O (0.0281 mg/L), and Na_2_MoO_4_·2H_2_O (0.024 mg/L) (Broughton and Dilworth 1971). For the test of salt stress, soybean seeds were grown in pots with autoclaved vermiculite (10 cm by 10 cm) and treated with 100 mM NaCl at sowing time. After emergence, three to five plants with similar growth vigour were kept, and others were removed. To boost the responsiveness of plants to salt stress, the soybean germplasms were treated with the same dose of NaCl for the second time after 11 days of sowing. The phenotypic traits were evaluated at 21 days after planting. Based on the response characteristics of soybean plants to salt stress, we selected one criterion—salt‐induced leaf senescence (SILS)—that can represent the salt tolerance of soybean seedlings. SILS was divided into 5 levels. Specifically, for soybean germplasm with only one trifoliate compound leaf, the classification criteria are as follows: (1) All the leaves wilted and died; (2) trifoliate compound leaves showed a wilting ranging from 0%–50%, and the unifoliate leaves died; (3) trifoliate compound leaves showed a wilting ranging from 0%–50%, and the unifoliate leaves showed a wilting ranging from 0%–50%; (4) trifoliate compound leaves grow normally without wilting, and the unifoliate leaves showed a wilting ranging from 0%–50%; (5) all leaves grow normally without wilting. For soybean germplasm with two or three trifoliate leaves, the classification criteria are as follows: (1) All the leaves wilted and died; (2) Only one pair of trifoliate compound leaves showed a wilting ranging from 0%–50%, and the rest of the leaves died; (3) two pairs of trifoliate compound leaves showed the wilting degree ranging from 0%–50%, and the rest of the leaves died; (4) the area of wilting of unifoliate leaves and the first pair of trifoliate compound leaves wilted ranged from 0%–50%, while the remaining leaves did not wilt; (5) all leaves grow normally without wilting. These two index systems were used for evaluating salt tolerance of wild soybean, landraces and cultivars, as well as subsequent GWAS.

### 4.2 Resequencing, Mapping and Variation Calling

Each library completed 150‐bp paired‐end reads (paired‐end sequencing) using an Illumina HiSeq X Ten device. As previously mentioned (Lu et al. 2020), resequencing data for the 1173 accessions was generated for the purposes of mapping and SNP calling. To put it briefly, BWA 0.6.1‐r104 software was used to map paired‐end reads to the Williams 82 (W82, Gmax_275_v2.0 from Phytozome) genome (Schmutz et al. 2010), using the default parameters (Li and Durbin 2009). SNPs and InDels were independently called using GATK (ver. 3.1.1) software (McKenna et al. 2010) and SAMtools (ver. 0.1.19) software (Li et al. 2009), and common sites identified using both methods were retained for further analysis. SNPs with missing data or with an MAF < 1% were removed, and InDels with a maximum length of 10 bp were included. According to the W82 genome, SNPs and InDels were annotated using ANNOVAR (ver. 2020‐06‐08) software (Wang et al. 2010). Synonymous and nonsynonymous SNPs were identified in coding sequences. In addition, we also conducted mapping and SNP calling in the Zhonghuang 13 genome, with parameters consistent with those mentioned above.

### 4.3 PCA

To conduct the PCA, SNPs of all soybean accessions were filtered with MAF > 5%. These SNPs were then analysed by PCA through TASSEL (Bradbury et al. 2007) and plotted by R software.

### 4.4 Linkage Disequilibrium Analysis

Using SNPs with MAF > 5%, linkage disequilibrium was computed for every subpopulation. To perform the linkage disequilibrium calculation, PLINK software (Purcell et al. 2007) was applied with the parameters (‐‐ld‐window‐r2 0 ‐‐ld‐window 99999 ‐‐ld‐window‐kb 1000). Based on the *r* ^2^ value between two SNPs, the decay of linkage disequilibrium was computed and averaged over 1 Kb intervals, with a maximum distance of 1 Mb.

### 4.5 GWAS for Salt Tolerance of Soybean

By utilising a total of 240 soybean accessions and 6 772 053 high‐quality SNPs (MAF > 5%), a GWAS was conducted to examine the salt tolerance of soybeans. Before being employed for genome‐wide or regional association analysis, DNA variations were quality screened using TASSEL, with the following requirements: a minimum minor SNP allele frequency of 0.05, a maximum proportion of heterozygous sites of 0.2, and a minimum number of accessions per site of 80%. Five principal components, as determined with TASSEL, were used as population structure (Q) matrices. Using TASSEL, the centred IBS approach was used to generate the kinship (K) matrix. A mixed linear model developed with TASSEL was used to do association analysis. *p* < 1.48 × 10^−7^ or −log_10_(*p*) > 6.83 is the number of markers; the threshold for a significant relationship was set at 1/*n*.

### 4.6 SV Identification

We extracted SVs' information from sorted and indexed BAM files using the delly (ver. 1.16) software (Rausch et al. 2012) with parameter ‘delly call ‐g ‐o’. SVs were divided into five types: duplications (DUP), insertions (INS), deletions (DEL), inversions (INV) and translocations (BND). We merged all BCF files using bcftools (ver. 1.18) (Li et al. 2009) to construct a nonredundant SVs set based on the Zhonghuang13 reference genome.

### 4.7 DNA Vector Construction and Soybean Hairy Root Transformation

To generate the construct harbouring *SST1* (or called *SST1* ^HapC^) fused with an MYC tag under the control of the CaMV35S (35S) promoter, the *SST1* coding sequence was amplified by PCR from Williams 82 genomic DNA and cloned into Pubi‐4MYC using *Asc* I. To generate the overexpression construct of *SST1* ^HapT^ with an MYC tag, the *SST1* coding sequence was amplified by PCR from Changnong 17 genomic DNA and cloned into Pubi‐4MYC using *Asc* I. A candidate target sequence of *SST1*, *Glyma.17G220100* and *Glyma.17G262700* for gene editing was identified using the web tool CRISPR‐P (http://crispr.hzau.edu.cn/CRISPR2/). The target sequence was subcloned into the single guide RNA (sgRNA) expression cassettes and transferred into the pKSE401 vector.

These constructs and the EVs were transformed into *A. rhizogenes* strain K599 for soybean hairy root transformation as described previously (Wang et al. 2014). The transgenic composite plants were transferred to small pots (7 cm by 7 cm) containing vermiculite and grown (16‐h light/18‐h dark, 25°C, 50% relative humidity). The composite plants overexpressing *SST1* were subjected to salt stress (100 mM NaCl) in distilled water at 10 days after transplanting. Phenotypic evaluation for salt tolerance was performed at 7 days after treatments. All soybean hairy root transformations were subjected to three independent experiments, with at least five plants involved in each experiment.

### 4.8 Nodulation and Symbiotic Nitrogen Fixation Analysis

To analyse symbiotic nitrogen fixation at mid and late stages under salt stress, WT and *sst1‐1* mutants were inoculated with USDA110 (30 mL USDA110; OD_600_ = 0.08) at sowing time and then treated with 100 mM NaCl 16 days after sowing, with 0 mM NaCl as control. The nodulation and symbiotic nitrogen fixation phenotype of WT and sst1 mutants were observed 9 days after salt treatment to evaluate their salt tolerance level under salt stress. Nodulation and symbiotic nitrogen fixation analysis were subjected to three independent experiments, with at least five plants involved in each experiment.

### 4.9 Promoter Analysis

According to the soybean genome information on the Phytozome website (Gmax_275_v2.0), the DNA sequence of the 3 kb promoter region upstream of the *SST1* start codon was input into the plantCARE (Lescot et al. 2002) database for promoter analysis. The *cis* elements in the *SST1* promoter were plotted using TBtools (Chen et al. 2023).

### 4.10 GUS Staining Assay

To make the *proSST1*:*GUS* construct, the promoter sequence of 2523 bp upstream of ATG, the start codon of *SST1*, was amplified from Williams 82 genomic DNA by PCR, and the promoter was then cloned into pCAMBIA1391‐GUS using *Hind* III and *EcoR* I. The *proSST1:GUS* construct was then transformed using a hairy root transformation system, and the composite plants were treated with or without 100 mM NaCl. The root samples were then collected at different time points and stained for 8 h with GUS staining (20 mM X‐Gluc). The stained hairy root samples were decolourised with 75% ethanol, and images of the samples were collected under a microscope. The soybean germplasm used is W82, as it contains full‐length SST1 protein.

### 4.11 Measurement of Growth Indicators and Physiological Analyses for Soybean Salt Tolerance

For the determination of root fresh weight, the whole root of soybean was cut off from the junction of root and stem, the vermiculite attached to the root was cleaned with clean water, and then the water on the surface of the root was sucked up with absorbent paper as far as possible, and finally the root was placed in a balance to measure the fresh weight. For the determination of leaf fresh weight, remove all soybean leaves and place them on a balance to measure the fresh weight. The contents of Na^+^ and K^+^ were determined using the pressure tank digestion method. Dry or fresh samples were weighed in the PTFE digestion inner tank and soaked overnight with 5 mL of nitric acid. The Na^+^ and K^+^ contents were analysed by the company (Wuhan Triploid Biotechnology Co. Ltd., Wuhan, China). The contents of proline, MDA and soluble sugar were measured using kit G0111W48, G0109W48, G0501W48 (Suzhou Grace Biotechnology Co. Ltd., Jiangsu, China), respectively. For the staining of H_2_O_2_, the soybean roots and leaves were soaked in DAB solution (coolaber, SL1805, 1 mg/mL, pH 3.8), incubated for 2 h, and then the samples were placed in 95% ethanol solution until they were decolourised and clarified. Finally, the sample was placed under a microscope to visualise the changes in H_2_O_2_. H_2_O_2_ contents were detected following the instructions of the Plant H_2_O_2_ ELISA Kit (mlbio, ml076343).

### 4.12 Western Blot Analysis of the SST1 Protein

The total protein of transgenic hairy roots expressing the EV and *pro35S*:*SST1*‐4MYC was extracted with protein extraction buffer (50 mM Tris–HCl, pH 7.5, 150 mM NaCl, 1 mM EDTA, 10% glycerol, 1% Triton X‐100, 1 mM PMSF and 1× protease inhibitor cocktail and 10 μM ΜG132). The samples were loaded equally onto an SDS‐PAGE gel and then blotted to a polyvinylidene fluoride membrane. The membranes were then probed with anti‐MYC antibody (SIGMA, C3956, 1:2000) or anti‐Actin (ABclonal, AC009, 1:2000) overnight. The soybean germplasm used is W82, as it contains the full‐length SST1 protein.

### 4.13 Generation of *sst1* Mutants

A candidate target sequence of *SST1* for gene editing was identified using the web tool CRISPR‐P (http://crispr.hzau.edu.cn/CRISPR2/). The target sequence was subcloned into the single guide RNA (sgRNA) expression cassettes and transferred into pBSE401 vector (Xing et al. 2014). Positive plasmids were introduced into *Agrobacterium tumefaciens* strain EHA105 for stable soybean transformation, which was performed according to a previously published protocol (Luth et al. 2015). The soybean germplasm used is W82, as it contains the full‐length SST1 protein.

### 4.14 RNA Extraction and Quantitative PCR Analysis

TRIpure Reagent (Aidlab Biotechnologies Ltd., Beijing, China) was used to extract total plant RNA. Using gDNA Wiper Mix (Vazyme Biotech Co. Ltd., Nanjing, China), genomic DNAs were eliminated. A FastQuant RT Kit (Vazyme Biotech Co. Ltd) was used to create cDNAs. SuperReal PreMix Plus (Vazyme Biotech Co. Ltd) was used to conduct qPCR studies. All assays were performed with at least three biological replicates. The primers for qPCR were listed in Table S11.

### 4.15 Subcellular Localization Analysis of the SST1 Protein

For subcellular localisation, the N‐terminal *SST1* fragment (Met1–Trp106) was amplified and inserted into the pB35S‐GFP‐BS‐2 vector because of the difficulty in the expression of the full‐length SST1 protein. Next, we fused the mitochondrial marker gene (*ScCOX4*) (Chen et al. 2019) with the mCherry tag to generate the construct driven by the CaMV35S (35S) promoter. The constructs were transformed into *A. tumefaciens* strain GV3101 for infiltration transformation of *Nicotiana* (*N*.) *benthamiana* leaves. GFP and RFP fluorescences were detected using a Leica confocal laser scanning microscope (Leica Microsystems).

### 4.16 RNA Editing Analysis of Mitochondrial Genes

Kobayashi et al.'s algorithm can accurately predict the targets of PLS‐PPR in *Arabidopsis*. We combined phylogenetic analysis of soybean and *Arabidopsis* to further predict the possible targets of SST1 (Kobayashi et al. 2019). For RNA editing analysis, 26 mitochondrial genes were amplified in WT and *sst1‐1* by RT‐PCR, and the PCR products were used for direct sequencing. The sequencing results of WT and the *sst1‐1* mutant were compared to evaluate changes in RNA editing status.

### 4.17 Transmission Electron Microscopy of Mitochondrial Morphology

Root tip sections of the WT and *sst1‐1* were collected and immersed in a fixative solution consisting of 5% glutaraldehyde in 100 mM phosphate buffer (pH 7.2). The samples were vacuum‐infiltrated for 10 min and transferred to an additional fresh fixative solution for 3 h at 4°C, then were rinsed with 0.1 mol/L PBS (pH 7.4) three times (15 min each) and fixed in 1% OsO4 in 0.1 mol/L phosphate buffer for 3 h at 22°C. The samples were rinsed in 0.1 mol/L PBS (pH 7.4) three times (15 min each) and then were dehydrated through a gradient ethanol series (50%, 70%, 80%, 90%, 95% and 100%) for 15 min, then subjected to three steps of ethanol/acetone (3:1, 1:1 and 1:3, each for 0.5 h) and to a final step of 100% acetone for 1 h. For infiltration, samples were treated with EMBed 812 (Haide, China) in three steps (with 1:1 acetone and EMBed 812, 2:1 acetone and EMBed 812, then pure EMBed 812).

### 4.18 Detection of Mitochondrial Function

The activity of complexes I, II, III, IV and ATP synthase was measured using kits G0845W48, G0846W48, G0847W48, G0848W48 and G0876W (Suzhou Grace Biotechnology Co. Ltd., Jiangsu, China), respectively.

### 4.19 Transcriptome Sequencing (RNA‐Seq) and Data Processing

The roots of both WT and the *sst1‐1* mutant plants were harvested to control treatment and salt stress (100 mM NaCl) treatment for 9 h at 15 d old for transcriptome analysis. RNA sequencing libraries were prepared from 2 μg of total RNA with the following modification. We removed ribosomal RNA using the Epicentre Ribo‐Zero rRNA Removal Kit. RNA sequencing was executed on the HiSeq 2500 platform (Illumina, San Diego, CA). One biological replicate was performed. Raw data underwent quality control and decontamination procedures utilising Trimmomatic (Bolger et al. 2014) to remove low‐quality and contaminated sequences.

The resulting clean data were aligned to the soybean reference genome (Gmax v4, Phytozome and NC_020455.1), employing the Hisat2 program (v2.1.0) (Kim et al. 2019) with default settings. The alignment files (SAM) were further refined using SAMtools (Quinlan and Hall 2010), applying a parameter (“‐q 30”) to eliminate reads with multiple hits, and were converted to BAM format. Reads count information was extracted from the BAM files for each RNA‐seq sample using the “multicov” function within the BEDTools program. To quantify gene expression levels, FPKM values were computed using the EdgeR program (Robinson et al. 2010). DEGs were identified using EdgeR, applying the criteria of a *q*‐value ≤ 0.05 and a foldchange in expression of ≥ 2. To pinpoint overrepresented GO terms among the DEGs in the *sst1‐1* mutant, we used the eggNOG‐mapper database (http://eggnog‐mapper.embl.de/) to create soybean‐specific GO terms based on the soybean reference annotation (Gmax v4, Phytozome). We subsequently subjected these designated categories to an analysis focusing on over‐represented GO terms. To assess the enrichment of GO terms, we carried out a hypergeometric test using the OmicShare tools (http://omicshare.com/tools).

### 4.20 Yeast Two‐Hybrid Assay

The full‐length coding sequences of *SST1*, *GmMORF1a* and *GmMORF1b* were amplified, and the PCR products were cloned into the entry vector pDONR207 by BP reactions. pDONR207 harbouring these genes was then ligated into pGBKT7 (BD) or pGADT7 (AD) by LR reactions. Yeast two‐hybrid (Y2H) assays were done according to the Matchmaker GAL4 Two‐Hybrid System (Clontech, Mountain View, CA).

For testing interactions, the above constructs were co‐transformed into the yeast strain *Saccharomyces cerevisiae* AH109. Transformation was confirmed by growth on SD/‐Leu/‐Trp (SD/‐2) medium. Interactions were assayed by spreading 2.5 μL of suspended transformed yeast on plates containing SD/‐Ade/‐His/‐Leu/‐Trp (SD/‐4) medium. The interactions were observed after 3–4 days of incubation at 30°C. The primers used in Y2H assays are listed in Table S1. Y2H assays were subjected to three independent experiments.

### 4.21 CoIP Assay

The SST1‐MYC construct was co‐expressed with the GmMORF1b‐HA construct in *N. benthamiana* leaves. Tobacco total proteins were extracted in extraction buffer (50 mM Tris–HCl, pH 7.5, 150 mM NaCl, 1 mM EDTA, 10% glycerol, 1% Triton X‐100, 1 mM PMSF and 1× protease inhibitor cocktail and 10 μM ΜG132) and then were incubated with 8 μL anti‐MYC agarose beads (SIGMA, A7470) for 2 h at 4°C. The beads were washed five times with extraction buffer, and the immunoprecipitated proteins were eluted with SDS loading buffer by boiling for 5 min for western blotting. Samples were analysed by western blot with anti‐HA (SIGMA, SAB4300603, 1:2500) and anti‐MYC (SIGMA, C3956, 1:2000).

### 4.22 Greenhouse Pot Experiments Under Different Salt Treatments

W82 and *sst1‐1* mutants in the W82 background were grown in a greenhouse (16 h:8 h, light:dark, 25°C, 50% relative humidity) in Wuhan (30°28′N, 114°21′E) in vermiculite. Soybean seeds were planted in pots (10 cm by 10 cm) containing autoclaved vermiculite. At least six biological replicates were designed for each plant. Each plant was treated with 0‰, 3‰, 6‰ and 9‰ NaCl after 14 days of sowing. Collect data on SILS, plant height, SPAD and the number of triple compound leaves for all plants on the 10th and 15th days of salt treatment.

### 4.23 Haplotype Analysis

SNPs and INDELs (MAF > 0.05) located in the gene body were extracted and subjected to haplotype analysis using the R package “geneHapR” (Zhang, Jia, and Diao 2023).

### 4.24 Molecular Evolution of *SST1* and *GmCHX1*

*F*_ST_ and nucleotide diversity for wild, landrace and cultivated populations of soybean were calculated using a 10 k–1 k sliding window and a 50 k–5 k sliding window in VCFtools (Danecek et al. 2011). The geographical information of soybean groups was acquired from Lu et al. (2020) and was marked on the map using R software to observe geographic distribution characteristics. The regions of saline soil were obtained from Liu and Wang (2021) and are presented in grey on the map. In order to evaluate the evolution of *SST1*, a phylogenetic tree and haplotype network were constructed using all the polymorphic sites with a minor allele frequency > 5% in this locus. The phylogenetic tree for *SST1* was constructed using the neighbour‐joining method by MEGA X (Kumar et al. 2018) and was visualised with the online tool Evolview v3 (https://www.evolgenius.info/evolview/), while the haplotype network was built by the PopART (Leigh and Bryant 2015). SNPs within the 0.5 Mb domestication region were used for sequence alignment analysis, which was performed as previously described (Goettel et al. 2022). All parameters of *GmCHX1*‐related evolutionary analysis were consistent with *SST1*.

### 4.25 Statistical Analysis

IBM SPSS Statistics 19, available from IBM Corp. in Armonk, NY, was used to analyse all of the data. Analysis of variance (ANOVA) and two‐sided *t*‐tests were used to produce *p* values. Graphs were created using R software and GraphPad Prism 9 (GraphPad Software).

## Author Contributions

X.L. designed the experiments. H.W., H.Y., Y.W., W.X., X.W., R.T., Q.X., J.L., and D.L. performed the experiments; Z.W., Q.Y., X.W., F.K. and B.L. conducted genome sequencing and data analysis of the natural population. H.W., Z.W. and X.L. wrote the manuscript.

## Conflicts of Interest

The authors declare no conflicts of interest.

## Supporting information


**Figure S1:** Evaluation of salt tolerance using SILS in soybean germplasm and QQ plot in GWAS of SILS.
**Figure S2:** The expression levels of three putative genes in response to salt stress.
**Figure S3:** Salt stress responses of transgenic soybean hairy roots overexpressing *Glyma.09G000400*, *Glyma.09G000700* and *Glyma.09G001200*.
**Figure S4:** Association analysis of two reference genomes.
**Figure S5:** SV‐GWAS manhattan plot of MLM model using ZH13 genome.
**Figure S6:** Bioinformatics analysis of the *SST1* gene and protein.
**Figure S7:** Relative expression analysis of *SST1* ^HapC/Hap3^ and *SST1* ^HapT/Hap2^ in the transgenic hairy roots overexpressing two haplotypes.
**Figure S8:** The genetic information of *sst1* mutant plants.
**Figure S9:** Phenotypic analysis of *sst1* mutants under salt conditions.
**Figure S10:** Phenotypic analysis of WT and *sst1‐1* at the V2 and V4 stages under salt treatment.
**Figure S11:** Nodulation phenotypes of *sst1* mutants under salt conditions.
**Figure S12:** Salt stress response in transgenic soybean hairy roots with knockout of *SST1* or its two homologues (*Glyma.17G220100* and *Glyma.17G262700*, referred to as *G1* and *G2*).
**Figure S13:** The expression pattern of *SST1* in leaves during salt stress.
**Figure S14:** Promoter analysis of the *SST1* gene.
**Figure S15:** GUS staining assay of the *SST1* promoter.
**Figure S16:** Prediction of SST1 subcellular localisation.
**Figure S17:** RNA editing of *cob*, *nad3* and *atp6‐1* and in hairy roots overexpressing *SST1* ^Hap3/HapC^or *SST1* ^Hap2/HapT^ and in Changnong 17.
**Figure S18:** Schematic diagram of sampling for electron transmission microscope experiment. The panel shows the sampling process and tissue location.
**Figure S19:** Electron transport chain activity and ATP synthase activity of WT and *sst1‐1* mutant.

**Table S1:** Distribution of subgroups and sources of 559 soybean accessions.

**Table S2:** The physical location and corresponding genes of potential candidate SNPs on soybean (Williams 82.a2) chromosome 9.

**Table S3:** Differentially transcribed genes derived from the RNA‐Seq experiment.

**Table S4:** Differentially transcribed genes derived from the RNA‐Seq experiment.

**Table S5:** GO enrichment of DEGs derived from the RNA‐Seq experiment.

**Table S6:** GO enrichment of DEGs derived from the RNA‐Seq experiment.

**Table S7:** FPKM of salt stress‐related genes in RNA‐seq.

**Table S8:** Differentially transcribed genes derived from the RNA‐Seq experiment.

**Table S9:** Differentially transcribed genes derived from the RNA‐Seq experiment.

**Table S10:** FPKM of mitochondrial genome DEGs in RNA‐seq.

**Table S11:** Primers used in this study.

## Data Availability

The data that supports the findings of this study are available in the Suppoting Information of this article.
