# Supplementary material for: Natural Variation of a PPR Coding Gene SST1 Confers Salt Tolerance During Soybean Domestication
Source: Plant Biotechnol J. 2025 Sep 25;24(2):831–55. doi: 10.1111/pbi.70382 (PMC12906803; doi:10.1111/pbi.70382)
Supplement: Supplementary file 1 — Figure S1: Evaluation of salt tolerance using SILS in soybean germplasm and QQ plot in GWAS of SILS. Figure S2: The expression levels of three putative genes in response to salt stress. Figure S3: Salt stress responses of transgenic soybean hairy roots overexpressing Glyma.09G000400, Glyma.09G000700 and Glyma.09G001200. Figure S4: Association analysis of two reference genomes. Figure S5: SV‐GWAS manhattan plot of MLM model using ZH13 genome. Figure S6: Bioinformatics analysis of the SST1 gene and protein. Figure S7: Relative expression analysis of SST1 HapC/Hap3 and SST1 HapT/Hap2 in the transgenic hairy roots overexpressing two haplotypes. Figure S8: The genetic information of sst1 mutant plants. Figure S9: Phenotypic analysis of sst1 mutants under salt conditions. Figure S10: Phenotypic analysis of WT and sst1‐1 at the V2 and V4 stages under salt treatment. Figure S11: Nodulation phenotypes of sst1 mutants under salt conditions. Figure S12: Salt stress response in transgenic soybean hairy roots with knockout of SST1 or its two homologues (Glyma.17G220100 and Glyma.17G262700, referred to as G1 and G2). Figure S13: The expression pattern of SST1 in leaves during salt stress. Figure S14: Promoter analysis of the SST1 gene. Figure S15: GUS staining assay of the SST1 promoter. Figure S16: Prediction of SST1 subcellular localisation. Figure S17: RNA editing of cob, nad3 and atp6‐1 and in hairy roots overexpressing SST1 Hap3/HapCor SST1 Hap2/HapT and in Changnong 17. Figure S18: Schematic diagram of sampling for electron transmission microscope experiment. The panel shows the sampling process and tissue location. Figure S19: Electron transport chain activity and ATP synthase activity of WT and sst1‐1 mutant. [file PBI-24-831-s006.docx]

**Supporting Figures S1-S19**


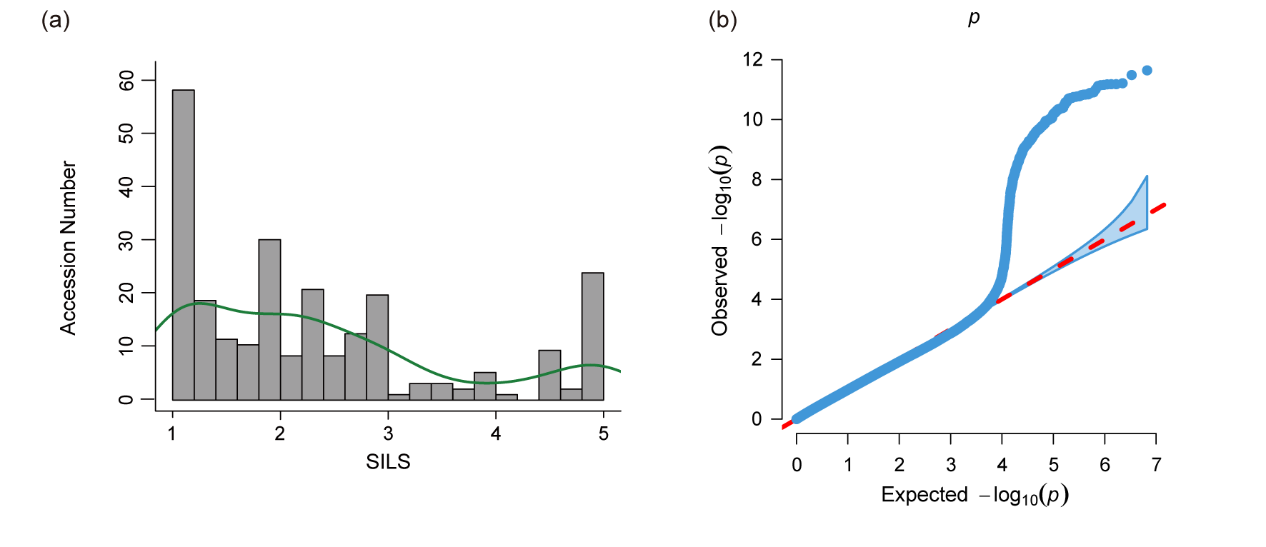
 **Figure S1** Evaluation of salt tolerance using SILS in soybean germplasm, and QQ plot in GWAS of SILS. (a) Phenotype distribution of SILS in soybean salt tolerance trait used for the association study. (b) QQ plot of SILS in soybean.


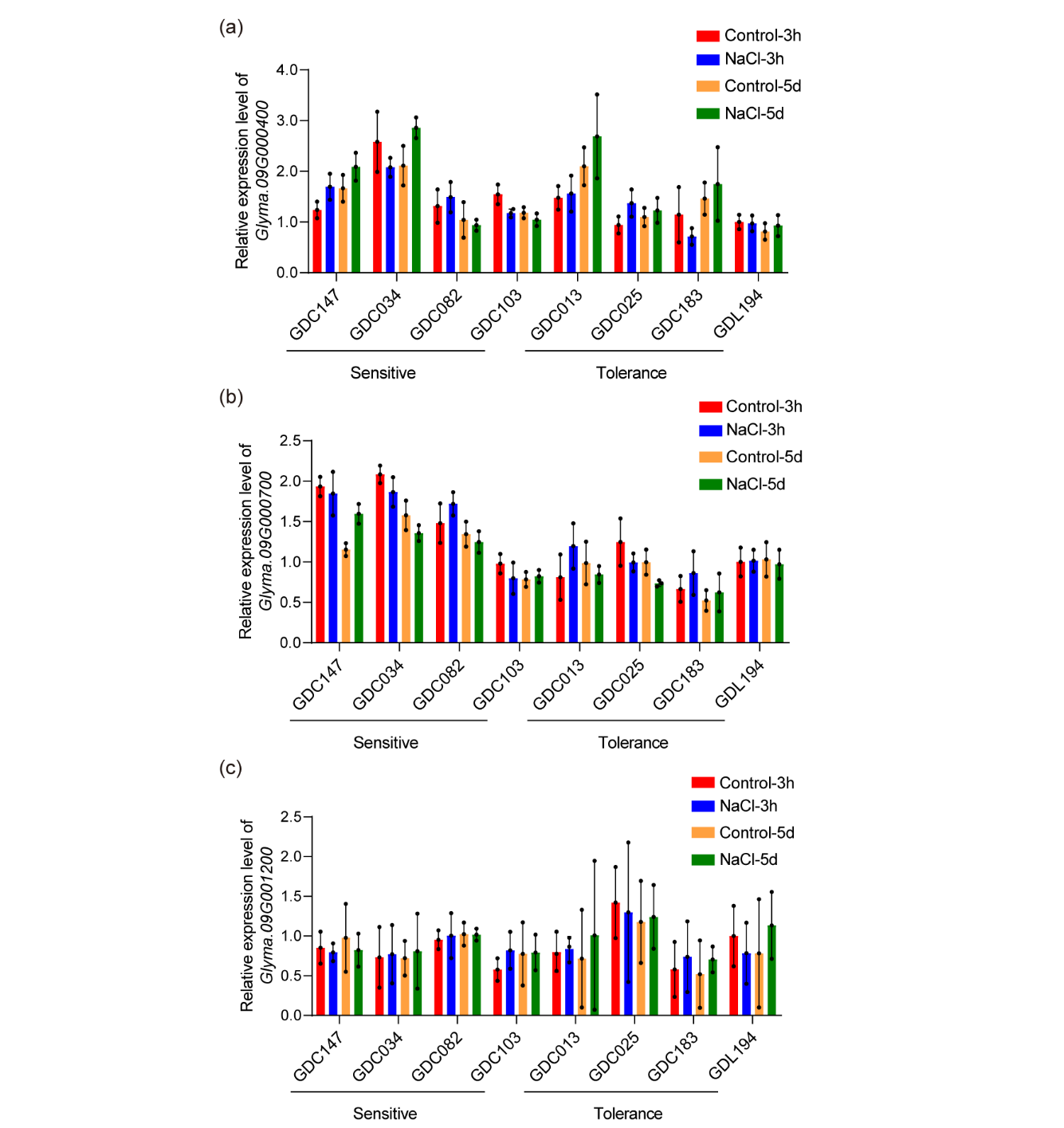


**Figure S2** The expression levels of three putative genes in response to salt stress. (a, b, c) Fourteen-day-old plants from randomly selected salt-tolerant and salt-sensitive accessions were treated either with or without 100 mM NaCl, and the transcript levels of *Glyma.09G000400*, *Glyma.09G000700* and *Glyma.09G001200* were analyzed at the indicated time points. Data shown are the means ± SD of three independent experiments. *GmELF1b* was used as the endogenous control gene. n = 3.


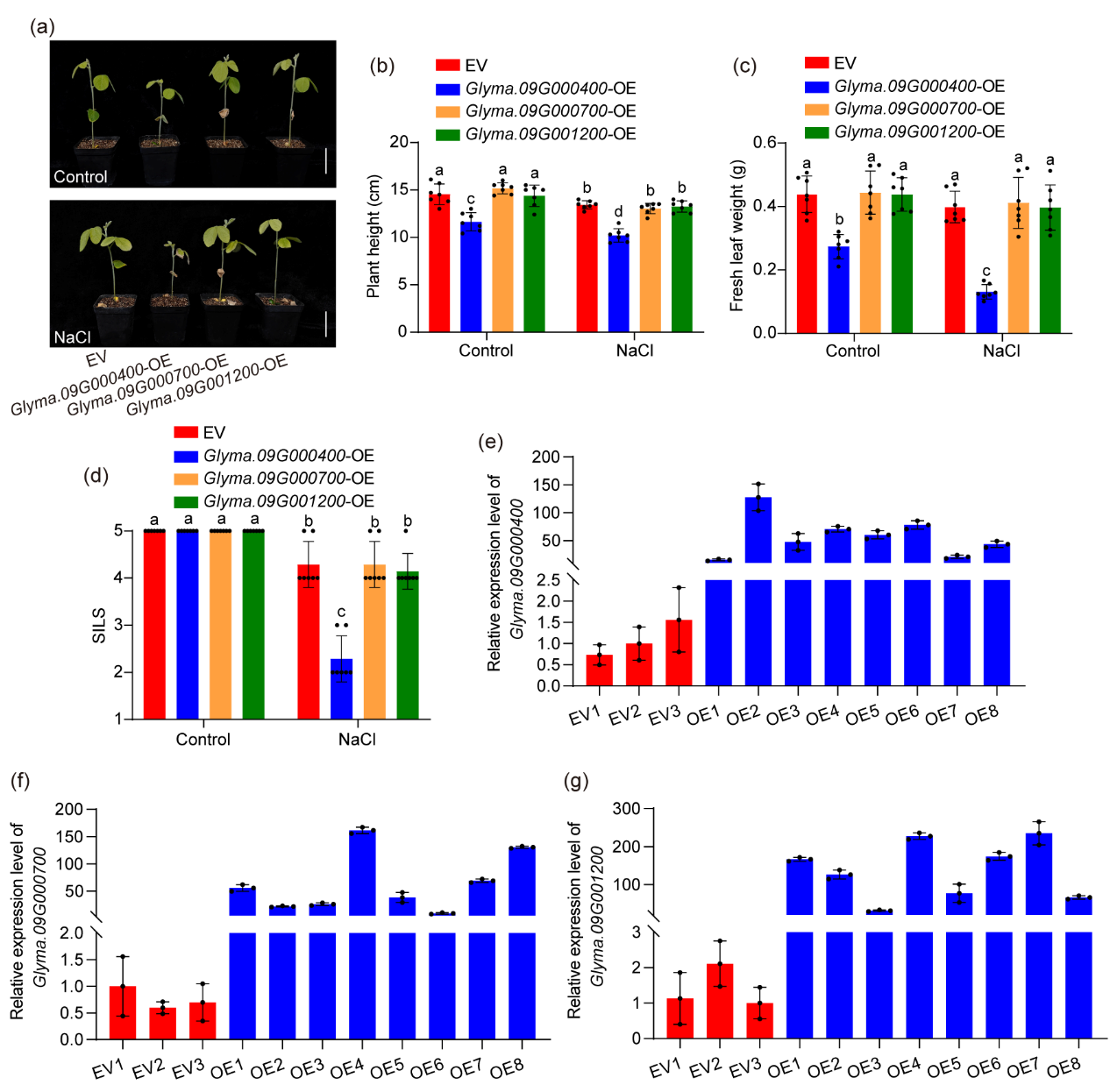
 **Figure S3** Salt stress responses of transgenic soybean hairy roots overexpressing *Glyma.09G000400, Glyma.09G000700* and *Glyma.09G001200*. (a) Phenotypes of *Glyma.09G000400, Glyma.09G000700* and *Glyma.09G001200* transgenic soybean hairy roots at control and NaCl (100 mM). Bars = 5 cm. (b, c, d) Comparison of plant height, fresh leaf weight and SILS of transgenic soybean hairy roots of *Glyma.09G000400*, *Glyma.09G000700* and *Glyma.09G001200* at control and NaCl (100 mM). Different letters indicate statistically significant differences at *P* < 0.05 by two-way ANOVA test. n=7. (e, f, g) Relative expression of *Glyma.09G000400*, *Glyma.09G000700* and *Glyma.09G001200* in empty vector and transgenic roots. n=3. Data shown are the means ± SD of three independent experiments.


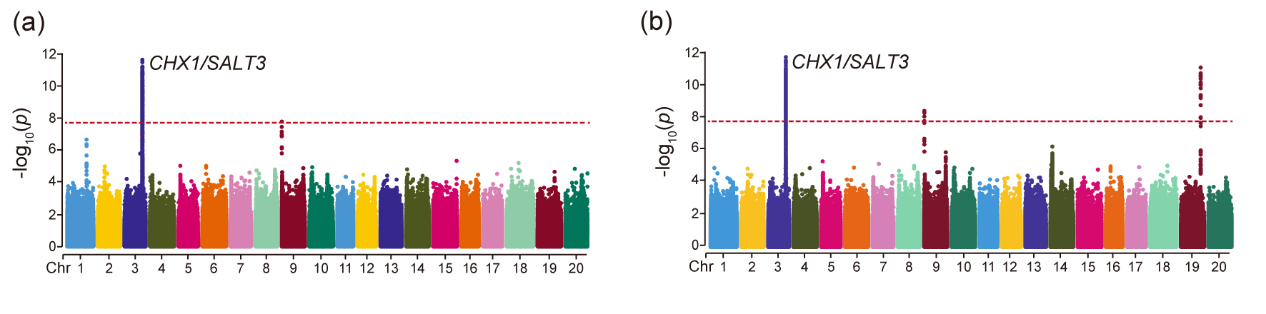


**Figure S4** Association analysis of two reference genomes. (a, b) Manhattan plots of W82.a2 genome (a) and ZH13 genome (b) using MLM model.


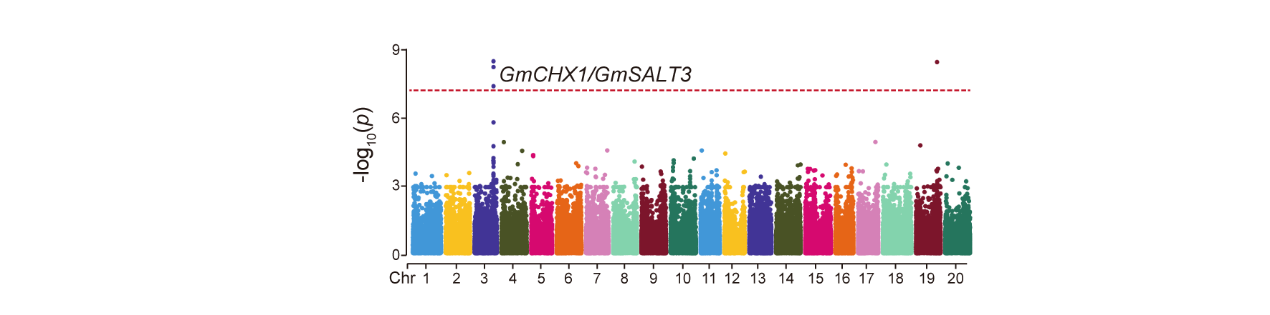


**Figure S5** SV-GWAS manhattan plot of MLM model using ZH13 genome.


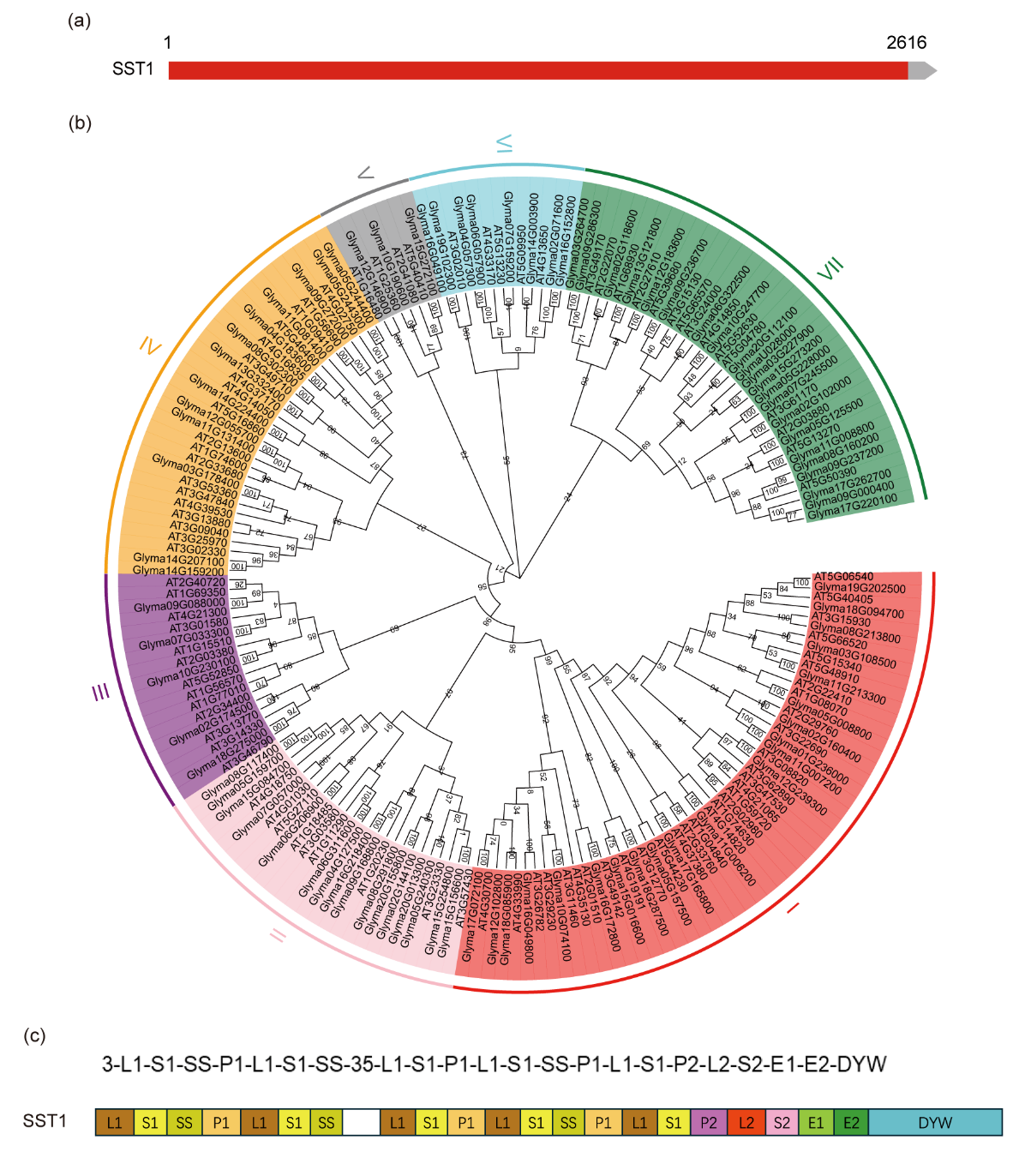


**Figure S6** Bioinformatics analysis of *SST1* gene and protein. (a) Schematic of *SST1* gene structure. (b) Phylogenetic tree of SST1 family proteins in soybean and *Arabidopsis*. (c) Identification of motifs in the SST1 protein sequence. The P motif is divided into P1 and P2 based on the first helix, P1 and P2 represent motifs encoding 35 amino acids; the L motif is divided into L1 and L2 based on the difference in the second helix, L1 and L2 represent long motifs encoding 35-36 amino acids; the S motif is also divided into the S1 motif composed of 31 amino acids and the S2 motif composed of 32 amino acids, while the SS motif represents a sequence that overlaps with both S1 and P1 motifs simultaneously.


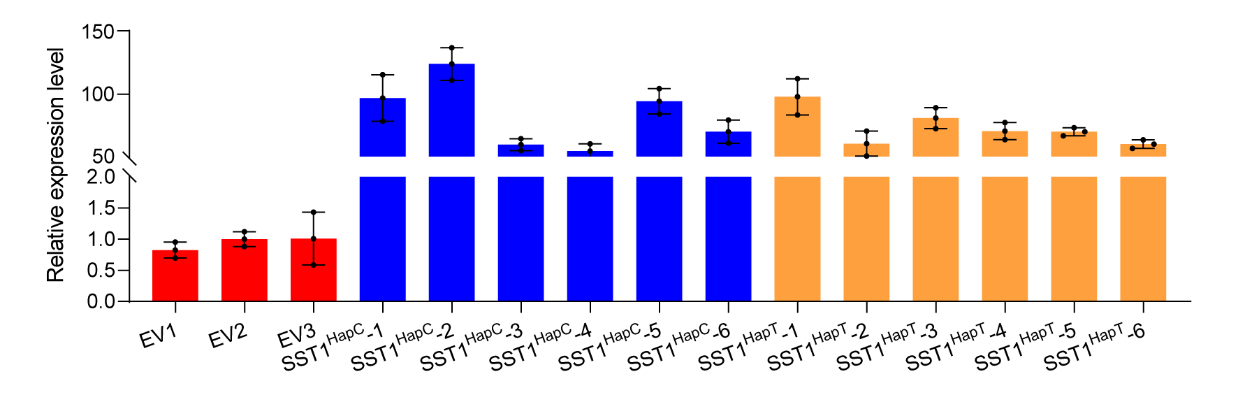
 **Figure S7** Relative expression analysis of *SST1*^HapC/Hap3^ and *SST1*^HapT/Hap2^ in the transgenic hairy roots overexpressing two haplotypes. The transgenic roots overexpressing *SST1*^HapC/Hap3^ (*SST1*^HapC/Hap3^-OE), *SST1*^HapT/Hap2^ (*SST1*^HapT/Hap2^-OE) and empty vectors were collected for RNA extraction and qPCR. *GmELF1b* was used as the internal reference control. n=3. Data shown are the means ± SD of three independent experiments.

**
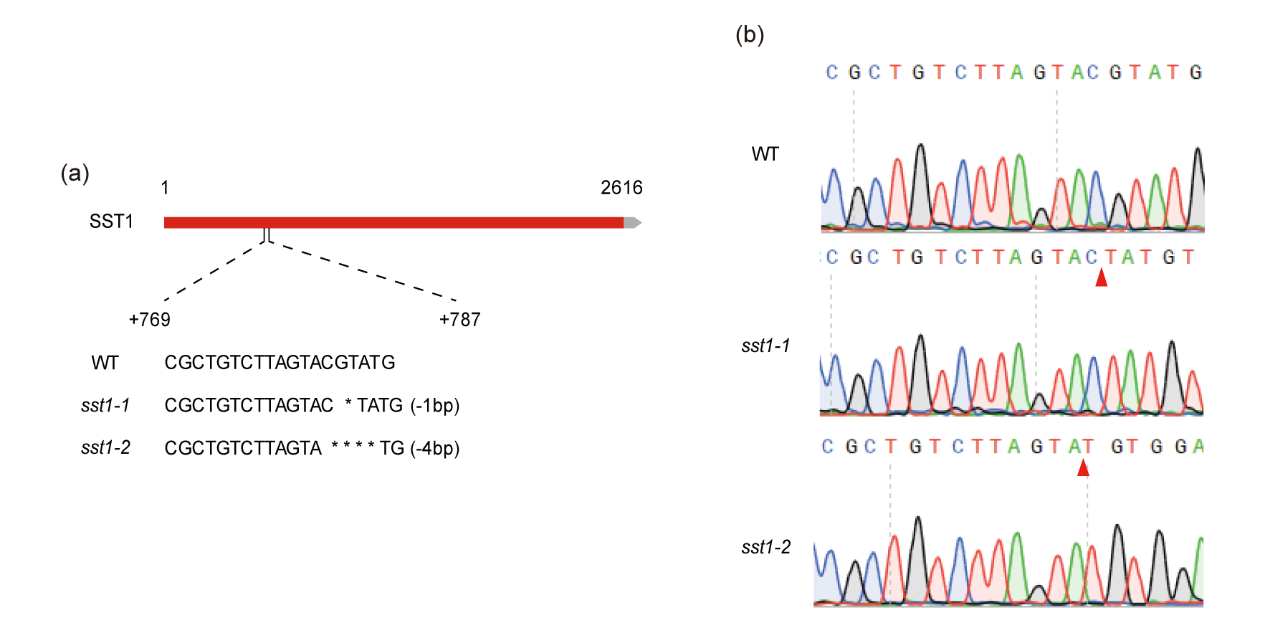
**

**Figure S8** The genetic information of *sst1* mutant plants. (a) Schematic diagram of gene editing of *sst1-1* and *sst1-2* mutants. The dotted line marks the target location in the mutants, and the asterisk represents the missing nucleotide bases. (b) Peak plot presentation of sequencing results for the wild type and *sst1* mutants. The red arrows point to target location in the mutants.


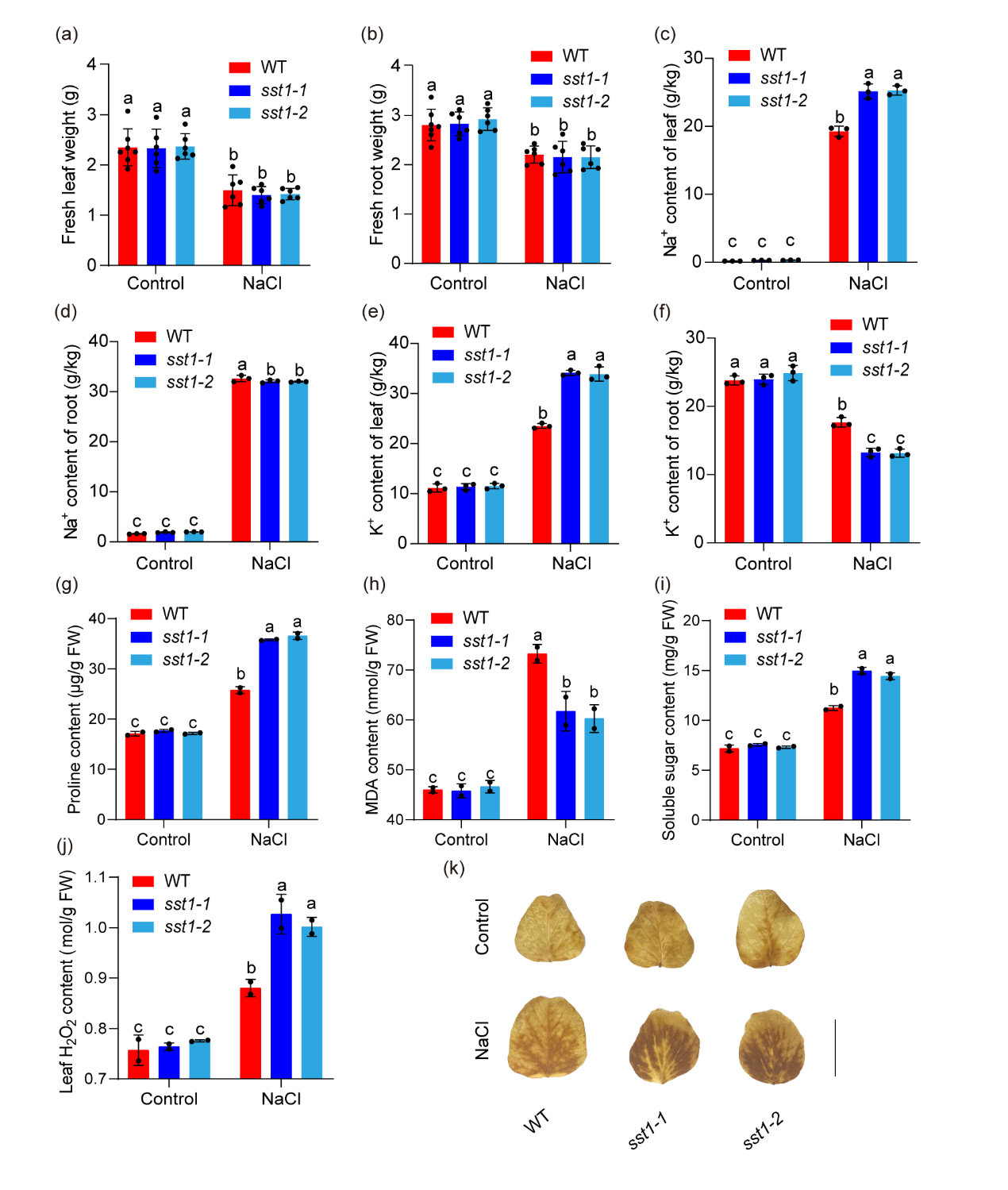


**Figure S9** Phenotypic analysis of *sst1* mutants under salt conditions. (a, b) Comparison of fresh leaf weight and fresh root weight of WT, *sst1-1* and *sst1-2* under control and NaCl (100 mM) conditions. Different letters indicate statistically significant differences at *P* < 0.05 by two-way ANOVA test (n = 7 for WT under control conditions; n = 6 for WT under 100 mM NaCl and for *sst1* mutants). (c, d, e, f,) Comparison of leaf Na^+^ content, root Na^+^ content, leaf K^+^ content and root K^+^ content of WT, *sst1-1* and *sst1-2* under control and NaCl (100 mM) conditions. Different letters indicate statistically significant differences at *P* < 0.05 by two-way ANOVA test. n = 3. (g, h, i) Comparison of proline (g), soluble sugars (h) and MDA content (i) of WT, *sst1-1* and *sst1-2* under control and NaCl (100 mM) conditions. Different letters indicate statistically significant differences at *P* < 0.05 by two-way ANOVA test (n = 2). (j) Comparison of leaf H_2_O_2_ content of WT, *sst1-1* and *sst1-2* under control and NaCl (100 mM) conditions. Different letters indicate statistically significant differences at *P* < 0.05 by two-way ANOVA test (n = 2). (k) DAB staining reveals the leaf H_2_O_2_ content of WT, *sst1-1* and *sst1-2* under control and NaCl (100 mM) conditions. The staining intensity reflects the concentration of H_2_O_2_. Bar = 3 cm.


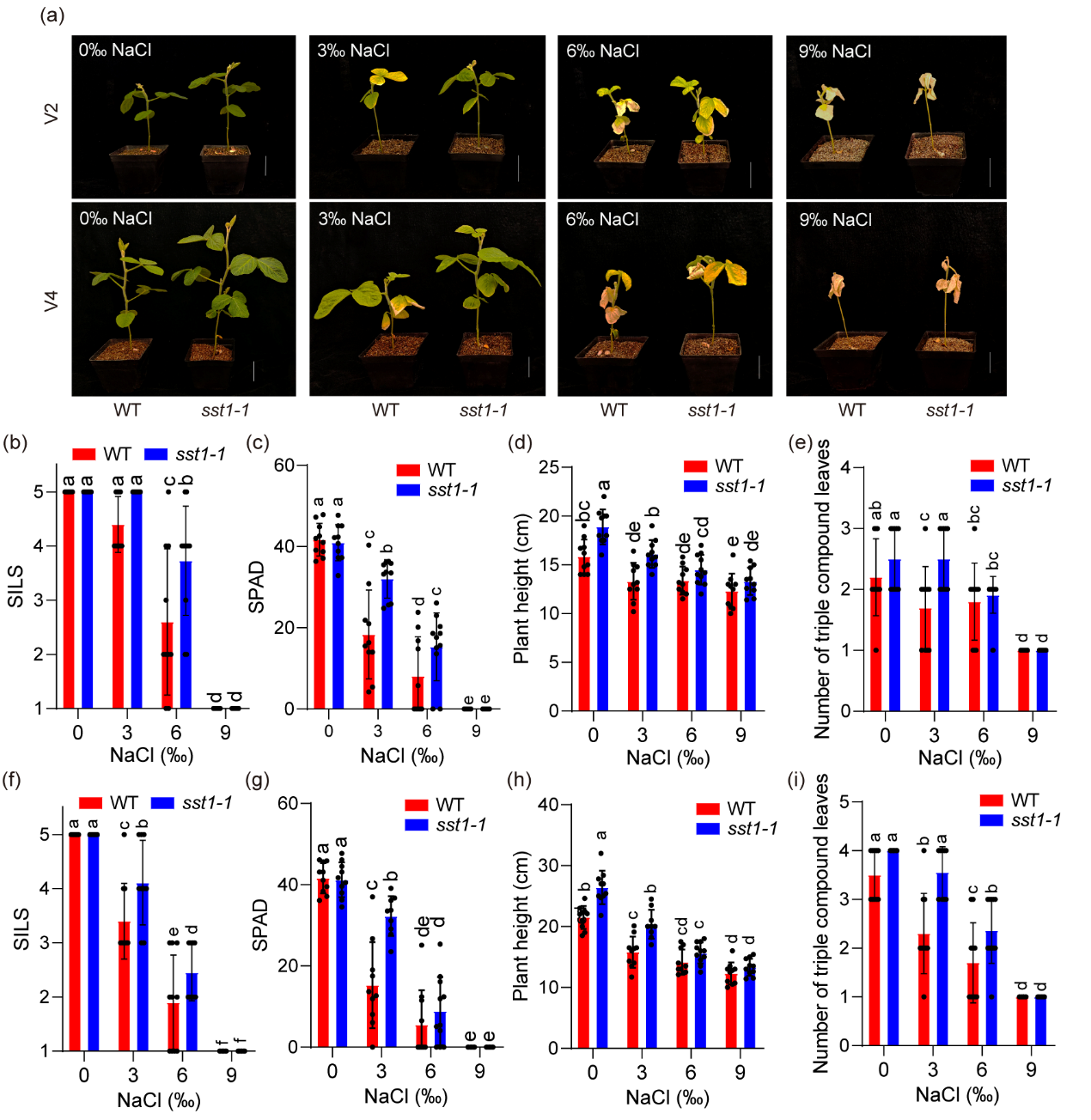


**Figure S10** Phenotypic analysis of WT and *sst1-1* at V2 and V4 stage under salt treatment. (a) Plant phenotypes of WT and *sst1-1* planted under 0, 3, 6 and 9‰ NaCl conditions. Bars=5 cm. (b, c, d, e) SILS, SPAD, plant height and number of triple compound leaves of WT and *sst1-1* at V2 stage under at 0, 3, 6 and 9‰ NaCl conditions. Bars=5 cm. (f, g, h, i) SILS (f), SPAD (g), plant height (h) and number of triple compound leaves (i) of WT and *sst1-1* at V4 stage under at 0, 3, 6 and 9‰ NaCl conditions. Bars = 5 cm. The significance of difference was analyzed by two-way ANOVA test, and different letters indicated the significance of difference (*P* < 0.05, n > 8).


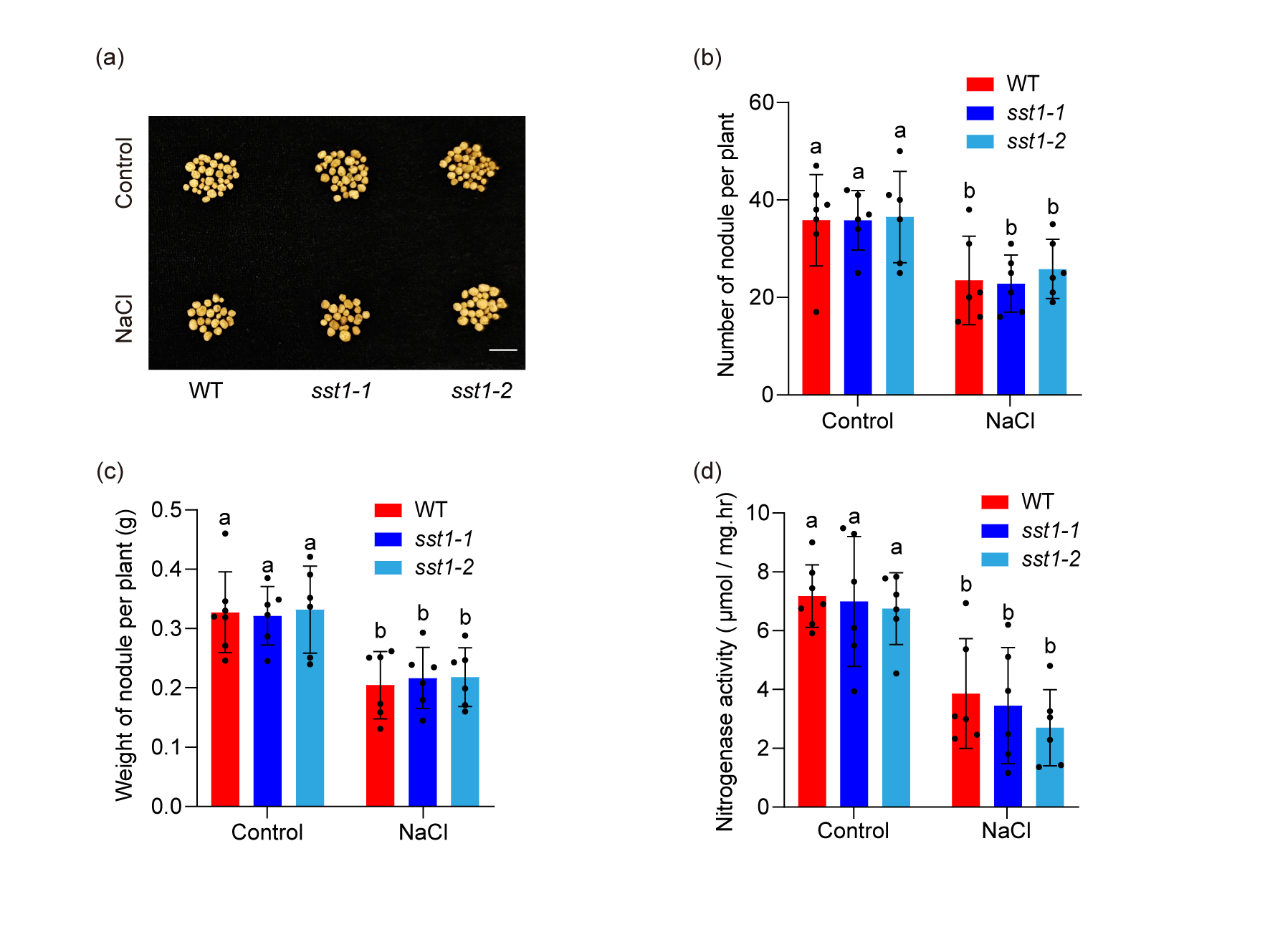


**Figure S11** Nodulation phenotypes of *sst1* mutants under salt conditions. (a) Nodule phenotype of WT, *sst1-1* and *sst1-2* plants under control and NaCl (100 mM) conditions. Bar = 1 cm. (b, c, d) Comparison of number of nodules per plant (b), weight of nodules per plant (c) and nitrogenase activity (d) of WT, *sst1-1* and *sst1-2* under control and NaCl (100 mM) conditions. Data shown are the means ± SD of three independent experiments. Different letters indicate statistically significant differences at *P* < 0.05 by two-way ANOVA test (n = 7 for WT under control conditions; n = 6 for WT under 100 mM NaCl conditions and for *sst1* mutants).


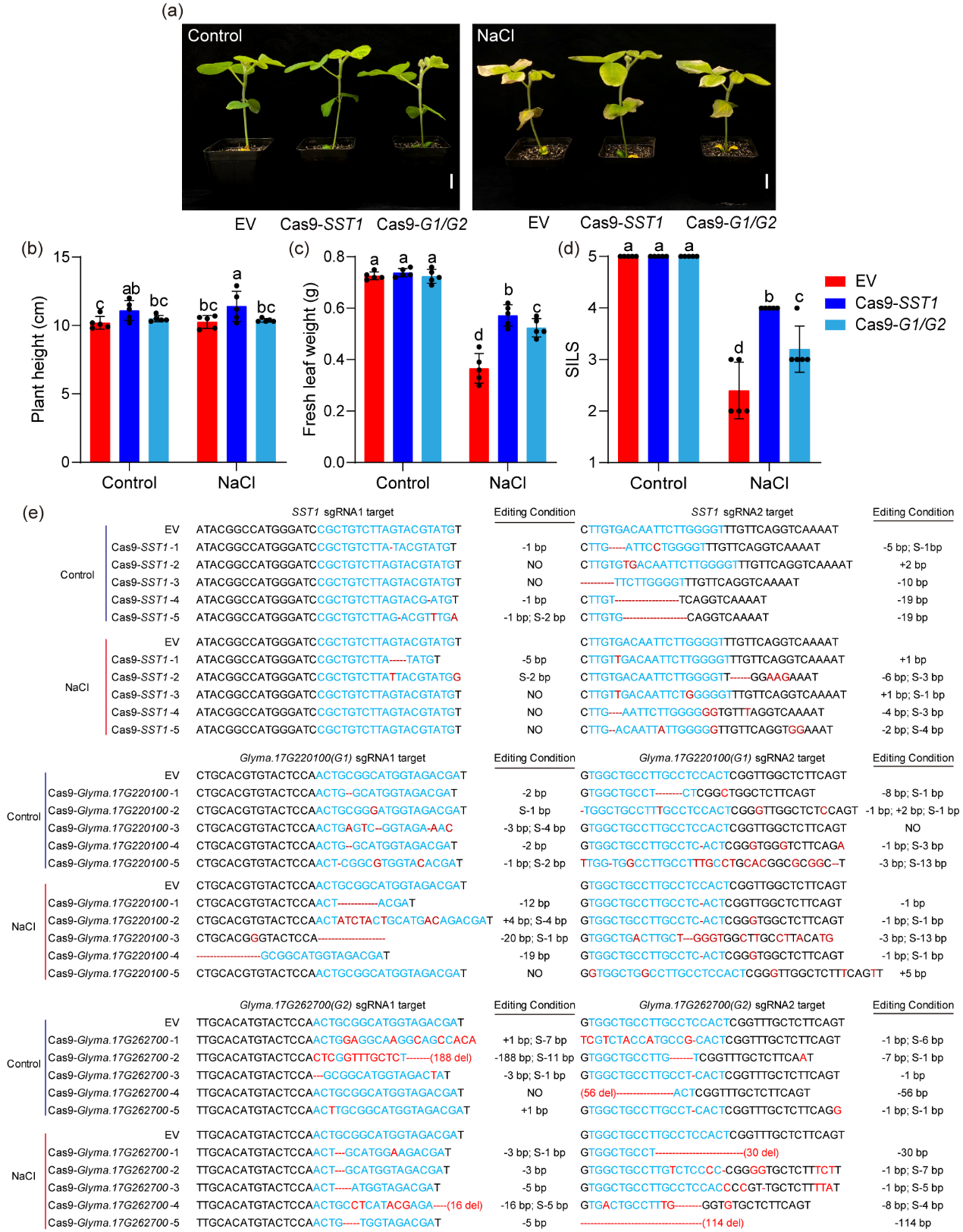


**Figure S12** Salt stress response in transgenic soybean hairy roots with knockout of *SST1* or its two homologs (*Glyma.17G220100* and *Glyma.17G262700*, referred to as *G1* and *G2*). (a) Phenotypes of *SST1* or *Glyma.17G220100* and *Glyma.17G262700* knockout transgenic soybean hairy roots under control and NaCl (100 mM) conditions. Bars = 2 cm. (b, c, d) Comparison of plant height (b), fresh leaf weight (c) and SILS (d) of transgenic soybean hairy roots of *SST1* and *Glyma.17G220100*, *Glyma.17G262700* knockout mutants under control and NaCl (100 mM) conditions. Different letters indicate statistically significant differences at *P* < 0.05 by two-way ANOVA test (n=5). (e) The gene editing conditions in the *SST1* and *G1*/*G2* knockout hairy roots. Each line in the bars indicates different gene editing condition in individual hairy roots. The letter "S" stands for base substitution, and "NO" stands for no editing occurs (n=5). Data shown are the mean ± SD of three independent experiments.


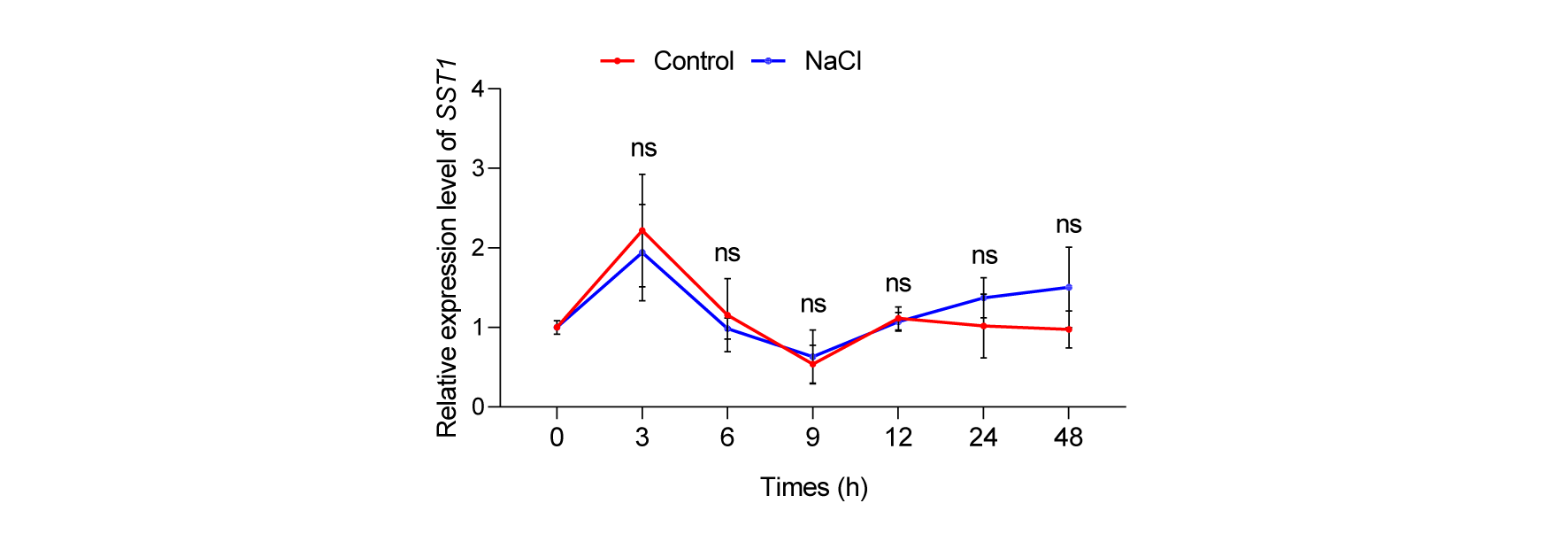


**Figure S13** The expression pattern of *SST1* in leaves during salt stress. Fourteen-day-old wild type seedlings were treated under control and NaCl (100 mM) conditions and leaf materials were collected at 0, 3, 6, 9, 12, 24 and 48 h after treatment to detect transcriptional level expression. *GmELF1b* was used as the endogenous control gene. Statistically significant differences were observed at a significance level of *P* < 0.05 using a two-sided *t*-test. n = 3. Data shown are the mean ± SD of three independent experiments.


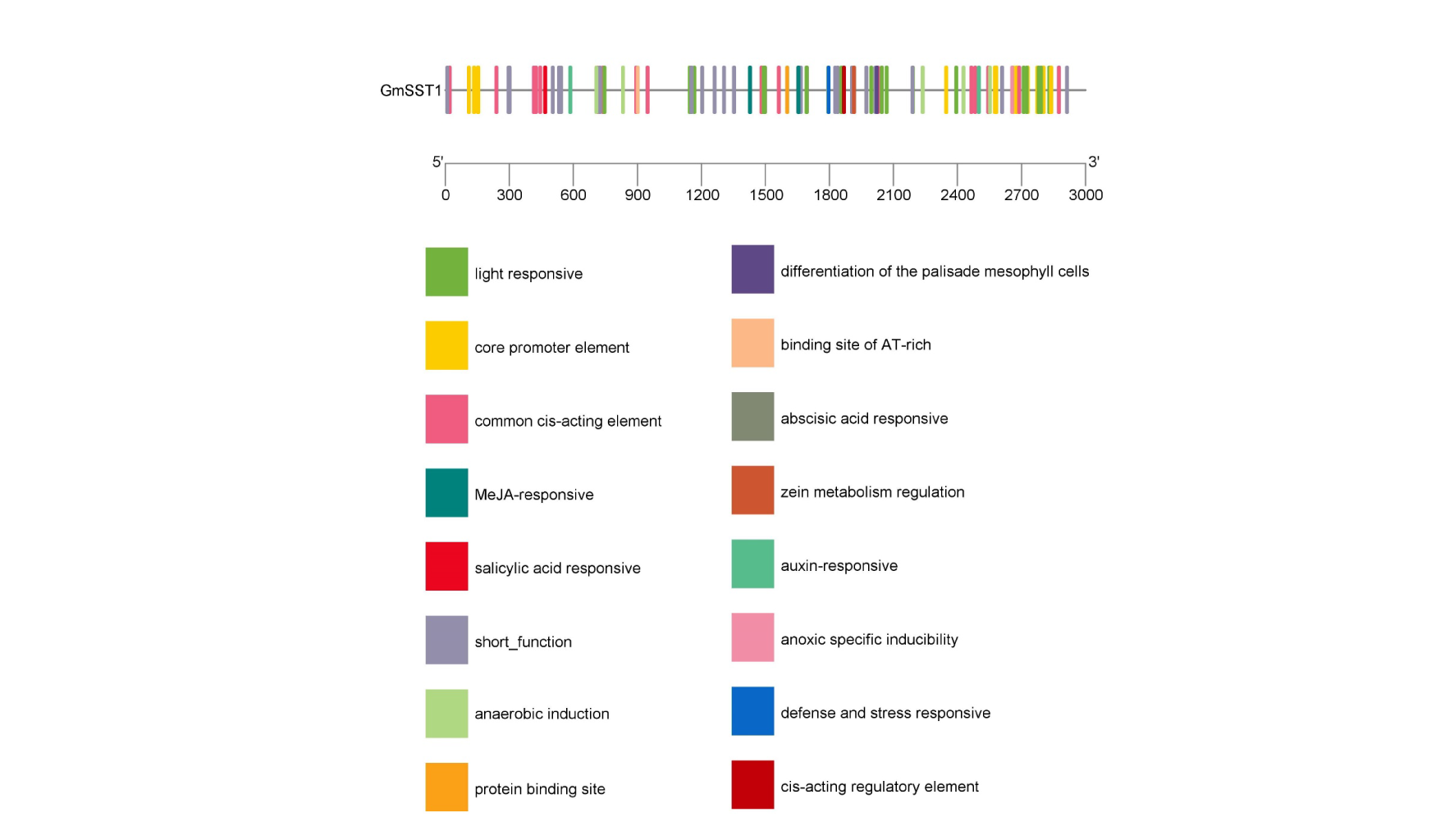


**Figure S14** Promoter analysis of the *SST1* gene. The promoter of the 3 Kb region upstream of the start codon of *SST1* was used to analyze the *cis* regulatory elements.


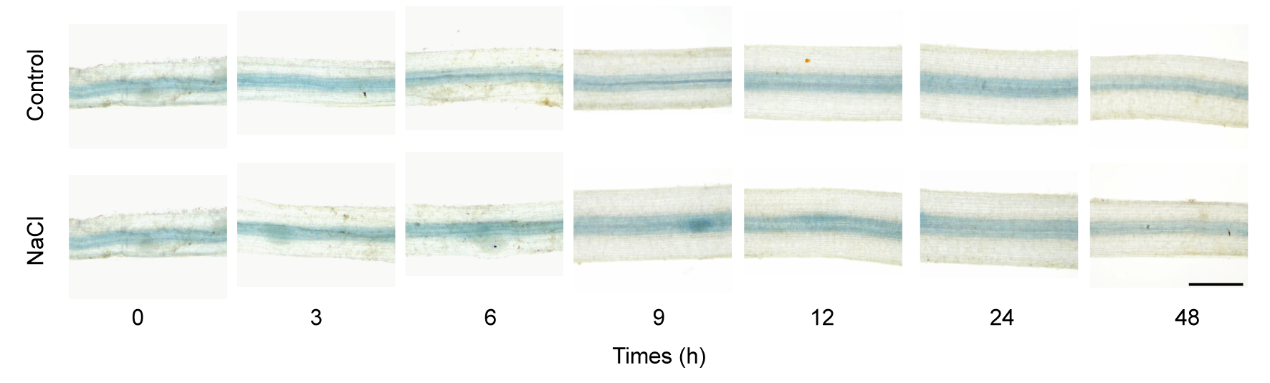


**Figure S15** GUS staining assay of the *SST1* promoter. The hairy roots expressing *proSST1:GUS* were treated with or without 100 mM NaCl, and were then harvested at 0, 3, 6, 9, 12, 24 and 48 h for GUS staining assay. The representative images of elongation regions of the *proSST1:GUS* roots were shown. Bars = 200 um, n=3.

**
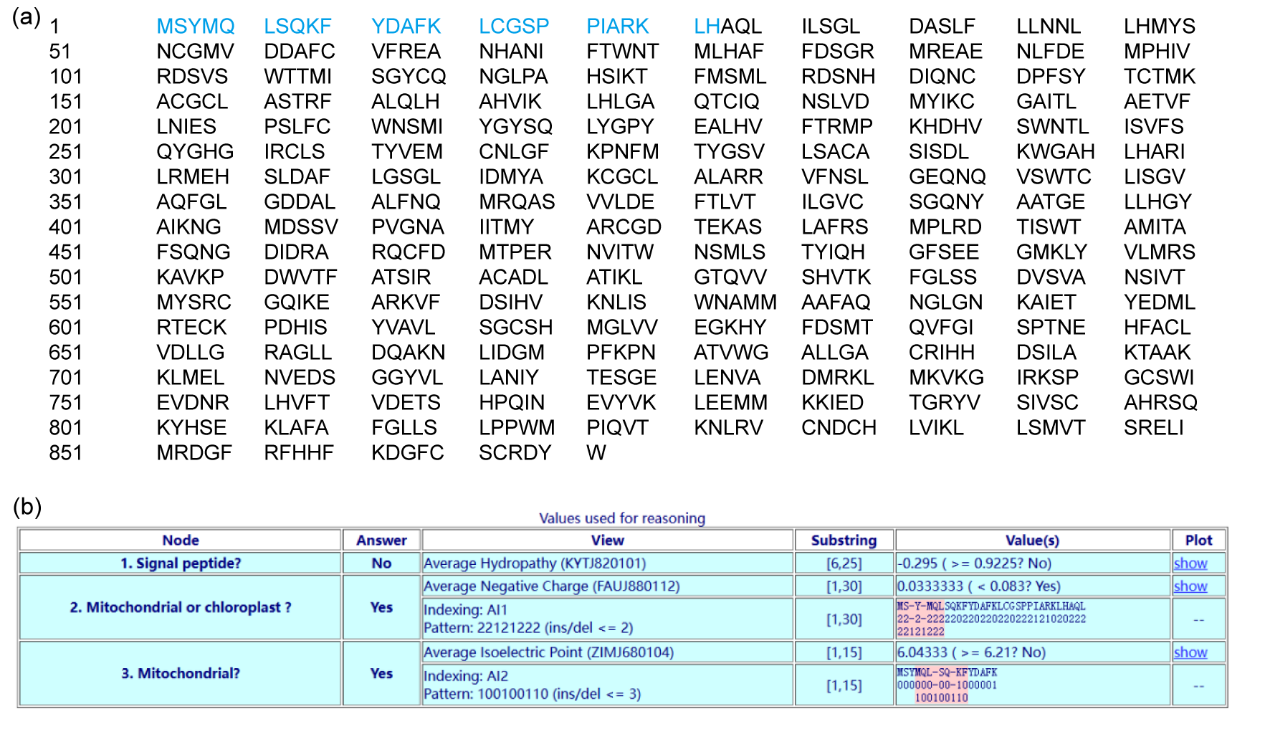
**

**Figure S16** Prediction of SST1 subcellular localization. (a) SST1 contains a mitochondrial targeting signal. The protein sequence of SST1 was analyzed using the iPSORT database, and the amino acids marked in blue were predicted as mitochondrial targeting peptide. (b) Prediction of subcellular localization of SST1. The amino acid sequence submitted to the iPSORT database undergoes subcellular prediction through three pipelines: Signal peptide, Mitochondrial or chloroplast, and Mitochondrial. The second column shows the predicted results, the fifth column shows the values.


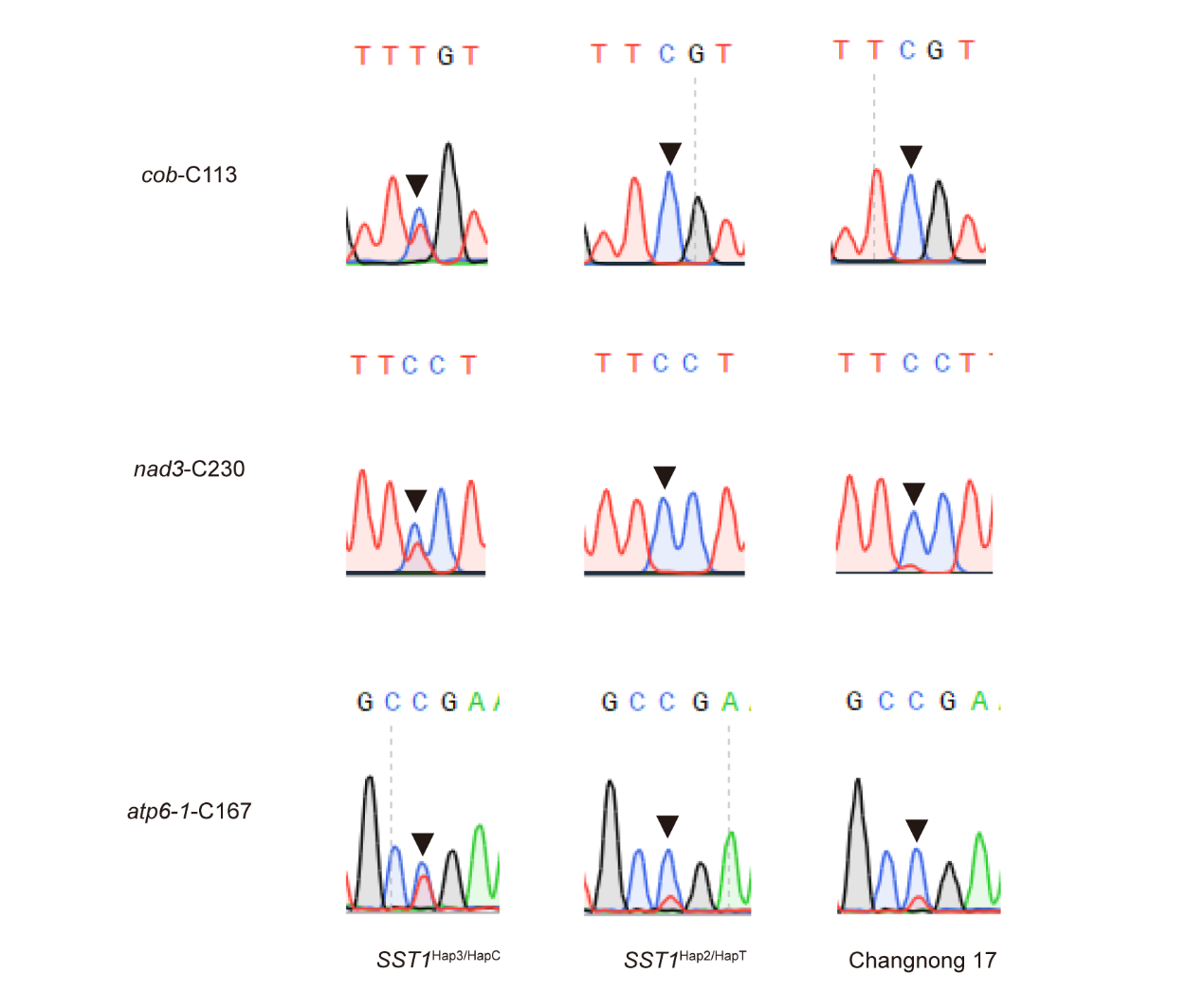


**Figure S17** RNA editing of *cob*, *nad3* and *atp6-1* and in hairy roots ovexpressing *SST1*^Hap3/HapC^or *SST1*^Hap2/HapT^ and in Changnong 17. RNA editing efficiency of the mitochondrial gene *cob*, *nad3* and *atp6-1* of *SST1*^Hap3/HapC^, *SST1*^Hap2/HapT^ and Changnong 17. The black arrows point to editing site.


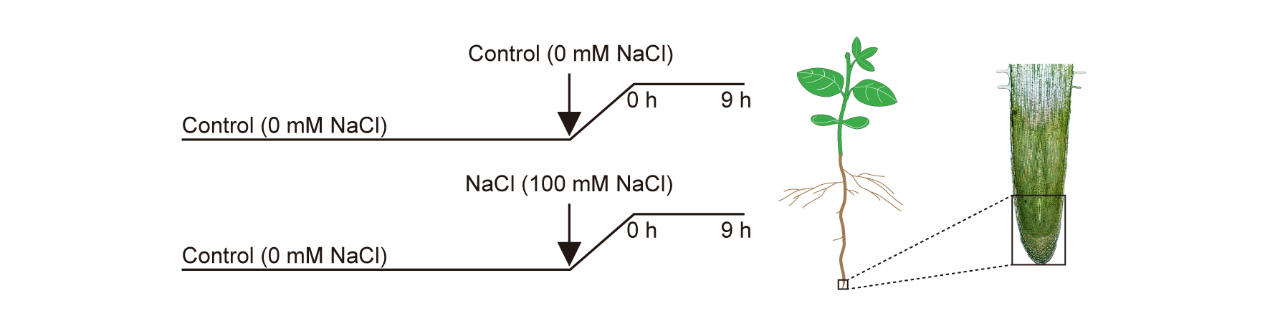


**Figure S18** Schematic diagram of sampling for electron transmission microscope experiment. The panel shows the sampling process and tissue location.


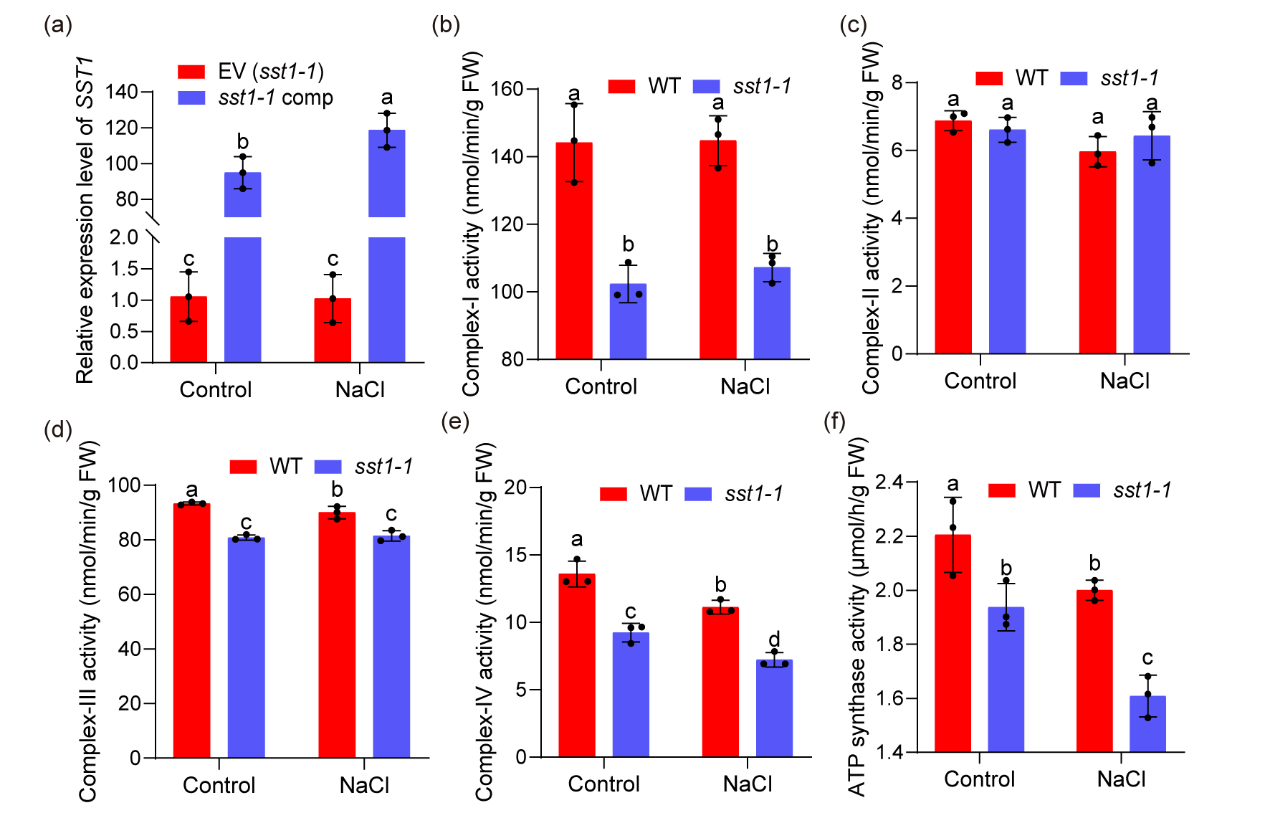


**Figure S19** Electron transport chain activity and ATP synthase activity of WT and *sst1-1* mutant. (a) Relative expression of *SST1* in empty vector and transgenic roots. n=3. (b, c, d, e, f) Comparison of complex I, II, III, IV and ATP synthase activity of WT and *sst1-1* under control and NaCl (100 mM) conditions. Different letters indicate statistically significant differences at *P* < 0.05 by two-way ANOVA test. n=3.
